# Mechanisms of VEGFR2 activation by VEGF, neuropilin, and heparin

**DOI:** 10.1126/sciadv.aeg6323

**Published:** 2026-07-15

**Authors:** Lianqi Chen, Zhichen Sun, Jian Qiao, Xiao-chen Bai, Xuewu Zhang

**Affiliations:** ^1^ Department of Pharmacology, University of Texas Southwestern Medical Center, Dallas, TX, USA.; ^2^ Department of Pathology, University of Texas Southwestern Medical Center, Dallas, TX, USA.; ^3^ Department of Biophysics, University of Texas Southwestern Medical Center, Dallas, TX, USA.; ^4^ Department of Cell Biology, University of Texas Southwestern Medical Center, Dallas, TX, USA.

## Abstract

Vascular endothelial growth factor (VEGF) and its receptor VEGFR are master regulators of vasculogenesis and angiogenesis. VEGF activates VEGFR by inducing its dimerization and trans-autophosphorylation. The coreceptor neuropilin (Nrp1 and Nrp2) and heparan sulfate proteoglycan (HSPG) modulate VEGF-VEGFR signaling, but the underlying mechanisms remain incompletely understood. Here we report a cryo-EM structure of the dimeric mouse VEGF_164_-VEGFR2-Nrp1 ectodomain complex with a 2:2:2 stoichiometry, revealing direct Nrp1-VEGFR2 interactions that stabilize the VEGFR2 dimer. We also determined two cryo-EM structures of the VEGF_164_-VEGFR2-Nrp1 complex in the presence of short- or long-chain heparin, which bridges all three proteins and promotes the formation of two distinct tetrameric complexes. Long-chain heparin induces a cis tetrameric complex consistent with receptor clustering on the same cell surface, whereas short-chain heparin promotes a trans tetrameric assembly which might be formed by two dimeric complexes from opposing cells. Our structure-based mutational analyses support the model that both the Nrp1-VEGFR2 interface and the heparin-mediated clustering enhance VEGFR2 signaling.

## Teaser

Cryo-EM structures reveal how the neuropilin and heparin stabilize VEGFR dimer and high-order oligomers for enhanced signaling.

## INTRODUCTION

Properly functioning blood vessels are essential for delivery of nutrients to and removal of metabolic wastes from tissues. Vascular endothelial growth factors (VEGFs) are the major regulators of vasculogenesis and angiogenesis (*1*). VEGFs act as angiogenic factors by activating the VEGF receptors (VEGFR1,2 and 3) expressed on the surface of endothelial cells. Signaling through the VEGF-VEGFR axis must be precisely regulated for the proper development and homeostasis of the vascular system. The importance of this regulation is underscored by the finding that genetic inactivation of a single allele of the *VEGF* gene is embryonically lethal (*2*, *3*). Dysregulation of the VEGF-VEGFR pathway is associated with many diseases, such as cancer, diabetic retinopathy, and age-related macular degeneration (*1*). Inhibition of VEGFR signaling by kinase inhibitors and antagonistic antibodies against VEGF or VEGFR has proven effective in treating these diseases in the clinic (*1*, *4*).

There are multiple isoforms of VEGF, as a result of alternative splicing and proteolytic cleavage (*5*–*7*). All VEGFs share the same N-terminal domain that forms a dimer, which binds and induces the dimerization of VEGFRs. VEGFRs belong to the receptor tyrosine kinase family, containing an extracellular region composed of seven immunoglobulin-like (Ig-like) domains, a single-transmembrane helix, an intracellular kinase domain, and a C-terminal tail with phosphorylation sites. The VEGF-induced dimerization of the VEGFR extracellular region promotes the activation of the kinase domain through trans-autophosphorylation, leading to phosphorylation of the C-terminal tail and recruitment of downstream signaling proteins that mediate mitogenic activity (*8*, *9*). VEGFR2 is the major angiogenic receptor for VEGFs, while VEGFR1, in some cases, can act as a “decoy” receptor that negatively regulates VEGFR2 signaling by competing for VEGF binding (*1*).

In addition to the common N-terminal domain, VEGF_165_ (VEGF_164_ in mouse), the most physiologically relevant VEGF isoform, contains two important features. The first is a heparin-binding domain (HBD) following the N-terminal domain, rich in positively charged residues that interact with negatively charged heparin and heparan sulfate proteoglycan (HSPG). The second is a C-terminal arginine residue, which mediates the interaction with the coreceptor neuropilin (neuropilins 1 and 2; referred to as Nrp1 and Nrp2 respectively thereafter) expressed on the cell surface together with VEGFRs (*5*, *10*–*12*). Both heparin and neuropilin enhance the activation of VEGFR by VEGF_165_, at least in part by facilitating the interaction between VEGF_165_ and VEGFR (*10*, *11*, *13*). VEGF_121_ is a weaker activator of VEGFR because it lacks the HBD (*7*, *14*). VEGF-Ax, featuring a 22-residue C-terminal extension, is less potent in inducing angiogenesis compared to VEGF_165_, likely because it does not have a C-terminal arginine residue and therefore shows impaired binding to neuropilin (*15*). Neuropilin has been shown to interact directly with VEGFR2 to form a receptor-coreceptor complex, which displays stronger signaling than VEGFR2 alone (*16*–*21*).

Nrp1 and Nrp2 are also single-pass transmembrane proteins, with short cytoplasmic tails that play roles in endocytic trafficking but have no major role in signaling (*22*–*26*). The N-terminal extracellular region of neuropilins contains five domains, named a1, a2, b1, b2, and c (also known as MAM) (*27*). Neuropilins also interact with heparin, through the positively charged surface patches in the b1 and b2 domains (*28*–*30*). Likewise, VEGFR2 has been shown to interact with heparin, but the binding site is not known (*31*, *32*). While VEGF_165_, VEGFR2, and neuropilin individually exhibit relatively weak binding to heparin, their combination has synergistic effects on enhancing the interaction with heparin and signaling (*33*).

The involvement of neuropilin and heparin in the VEGF-VEGFR complex makes the activation mechanism of VEGFR more complex than the general ligand-induced dimerization paradigm for the activation of receptor tyrosine kinases. While previous structural studies have resolved the mechanism of VEGF-induced dimerization of VEGFR (*8*, *9*, *34*), critical mechanistic questions—such as how neuropilin interacts with VEGFR and how heparin enhances the formation of the VEGF-VEGFR-neuropilin complex and its signaling—remain open. In this study, we address these questions by presenting three cryo–electron microscopy (cryo-EM) structures of the mouse VEGF_164_-VEGFR2-Nrp1 complex, with or without heparin bound. These structures together with structure-based mutational analyses reveal previously unknown mechanisms that underlie the cooperative effects of neuropilin and heparin in VEGFR2 activation.

## RESULTS

### Structure of the VEGF_164_-VEGFR2-Nrp1 complex

**Fig. 1.**
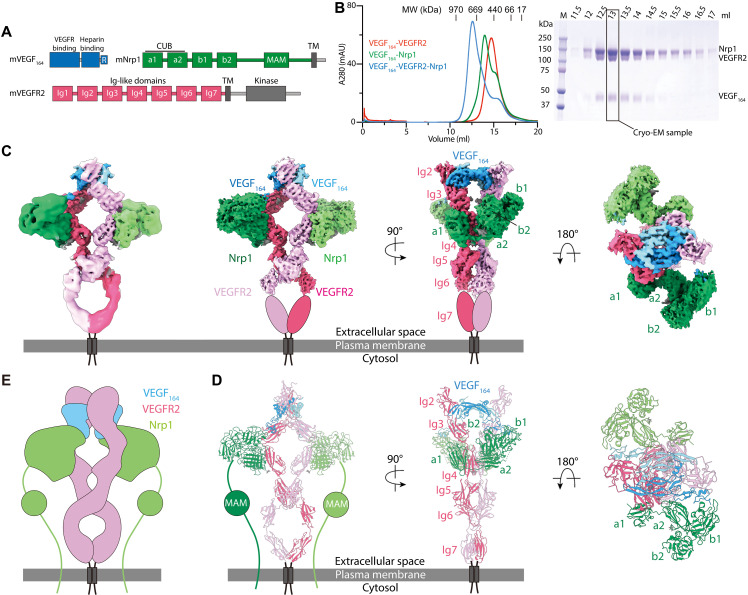
Cryo-EM structure of the VEGF_164_-VEGFR2-Nrp1 dimeric complex. (**A**) Domain structures of mouse VEGF_164_, VEGFR2, and Nrp1. Colored parts were included in the constructs for the structural analyses. TM, transmembrane. (**B**) Profiles of the VEGF_164_-VEGFR2-Nrp1 complex compared to the VEGF_164_-Nrp1 and VEGF_164_-VEGFR2 complexes from a Superose 6 size exclusion column. A280, absorbance at 280 nm; mAU, mini-absorbance unit. The right panel shows the SDS–polyacrylamide gel electrophoresis (SDS-PAGE) analyses of the fractions of the VEGF_164_-VEGFR2-Nrp1 complex. MW, molecular weight. (**C**) Overview of the cryo-EM map of the VEGF_164_-VEGFR2-Nrp1 complex. The middle and right show the map used for structural refinement, while the left shows a low-pass filtered map contoured at a low threshold to illustrate the presence of VEGFR2-Ig7 in the density. (**D**) Atomic model of the VEGF_164_-VEGFR2-Nrp1 complex based on the cryo-EM reconstruction. (**E**) Schematic model of the dimeric complex based on the cryo-EM structure. The intracellular kinase domain of VEGFR2 is omitted in the diagrams in (C) to (E) for simplicity.

To understand how neuropilin contributes to the activation of VEGFR2 by VEGF, we reconstituted the ectodomain complex of purified mouse VEGF_164_, VEGFR2 (residues 20 to 760), and Nrp1 (residues 22 to 855) (Fig. 1A). The size exclusion chromatography (SEC) results showed that the three proteins together formed a complex larger than either of the two-protein complexes of VEGF_164_-VEGFR2 and VEGF_164_-Nrp1 (Fig. 1B). We solved the cryo-EM structure of the VEGF_164_-VEGFR2-Nrp1 complex to an overall resolution of 3.3 Å (Fig. 1C and fig. S1). The atomic model based on this map contains most regions of the three proteins, except for Ig1 of VEGFR2 and the segment in Nrp1 C-terminal to the b2 domain. The density for Ig7 in VEGFR2 is weak but clearly visible in the low-pass filtered map displayed at a low threshold (Fig. 1C).

The VEGF_164_-VEGFR2-Nrp1 complex displays a symmetric dimeric structure, with a 2:2:2 stoichiometry, composed of one VEGF_164_ dimer (two VEGF subunits) associated with two VEGFR2 and two Nrp1 molecules (Fig. 1, C and D). Consistent with the previous structures, the VEGF_164_ dimer uses its N-terminal domain to bind the Ig2 and Ig3 domains of VEGFR2 and bring two VEGFR2 protomers into proximity for the dimerization and activation (*8*, *34*, *35*). The six Ig-like domains of VEGFR2 visible in the structure adopt an overall “S” shape similar to VEGFR1 (fig. S2A) (*8*). The two VEGFR2 molecules arranged in a parallel fashion make intermolecular interactions through their Ig4, Ig5, and Ig7 domains analogous to those seen in the VEGF-VEGFR1 structure. These interactions have been shown to contribute to the signaling by bringing the membrane proximal regions close and thereby promoting the activation of the kinase domain (*8*). These features of the VEGF and VEGFR complex have been well characterized by previous studies, and therefore we will not discuss them further.

Our cryo-EM map clearly shows two Nrp1 molecules bound to the two sides of the VEGF_164_-VEGFR2 dimeric complex (Fig. 1, C and D). The a2-b1-b2 domains of Nrp1 form a stable structural module with a characteristic triangular shape as seen previously (*27*, *29*, *36*). The vertex of the triangle formed by the a2 domain interacts with the Ig4 domain in VEGFR2, while the vertex formed by the b1 domain makes contacts with the HBD in VEGF_164_ (Fig. 1, C and D). The C-terminal arginine residue of VEGF_164_ engages the ligand-binding pocket in the b1 domain of Nrp1, as expected from previous studies (*12*). In addition, the HBD of VEGF_164_ and Nrp1-b1 makes a small domain-domain interface similar to the previous crystal structure of the VEGF/Nrp1 complex (fig. S2F) (*12*). The overall binding mode between VEGF_164_ and Nrp1 is well supported by the cryo-EM, although the details of the interface are not resolved due to the weak density of this area. In addition to a2, the a1 domain in Nrp1 forms a binding interface with the Ig4 domain of VEGFR2 (Fig. 1, C and D). The three anchoring interactions formed by the a1, a2, and b2 domains in Nrp1 with VEGF_164_ and VEGFR2 collectively stabilize the 2:2:2 complex (Fig. 1E). These interactions likely underlie the positive effects of neuropilin on VEGF-stimulated VEGFR signaling (*16*–*21*). The MAM domain and the long linkers flanking the MAM domains are not resolved in the cryo-EM map. While this region of Nrp1 is not engaged in any protein-protein interaction, it serves as a linker to span the distance between the b2 domain and the transmembrane region of Nrp1, similar to that in the Sema3a/PlexinA4/Nrp1 complex (Fig. 1D) (*27*).

### Binding interface between VEGFR2 and Nrp1

**Fig. 2.**
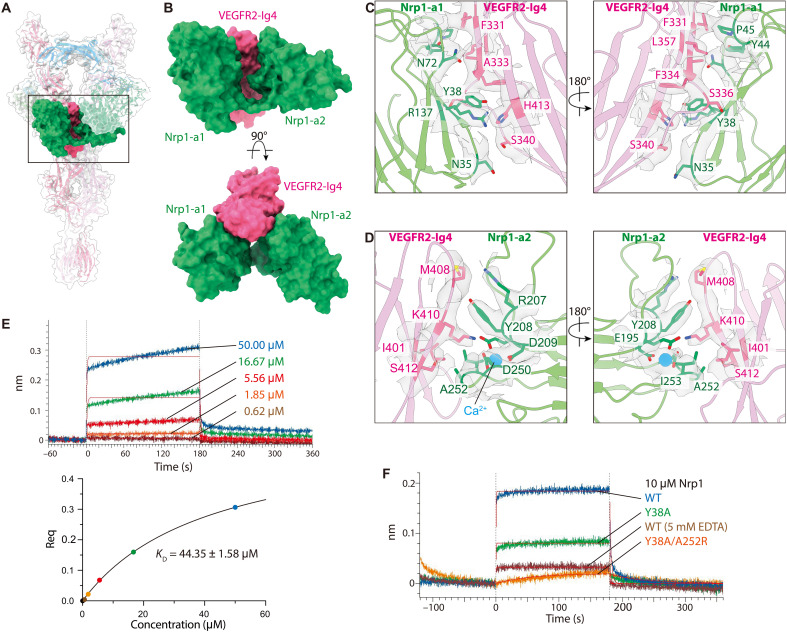
Binding interface between VEGFR2 and Nrp1. (**A**) Overview of the binding mode between VEGFR2-Ig4 and Nrp1-a1-a2 in the dimeric complex. (**B**) Surface rendering of the interaction between VEGFR2-Ig4 and Nrp1-a1-a2 in two different views. (**C**) Close-up view of the interface between VEGFR2-Ig4 and Nrp1-a1. Cryo-EM density at the binding interface is shown in semitransparent gray. (**D**) Close-up view of the interface between VEGFR-Ig4 and Nrp1-a2. Cryo-EM density at the binding interface is shown in semitransparent gray. (**E**) Biolayer interferometry (BLI) analysis of the interaction between VEGFR2 and Nrp1. Binding of Nrp1 at the indicated concentrations to biotinylated VEGFR2 immobilized on a streptavidin-conjugated sensor was measured. The bottom shows the results of the steady-state analysis using a 1:1 binding model. Req, response at the equilibrium phase. (**F**) Mutations of Nrp1 at the binding interface (Y38A and Y38A/A252R) or removal of calcium ion by EDTA reduce the binding to VEGFR2. WT, wild type.

Our cryo-EM structure provides a basis for the direct interaction between VEGFR2 and Nrp1 suggested by previous studies (*18*, *20*, *21*). The interaction is mediated by the a1 and a2 domains in Nrp1, which together form a clamp-like structure to grasp the outer surfaces of the Ig4 β-sandwich in VEGFR2 (Figs. 1D and 2, A and B). The interface buries a total solvent accessible area of ~1970 Å^2^. The a1 and a2 domains in Nrp1 also interact with plexin and semaphorin in the Sema3A-PlexinA4-Nrp1 complex, although the two domains adopt a different relative orientation in that case (fig. S2, C and D) (*27*). Superimpositions of the structure of the VEGF_164_-VEGFR2-Nrp1 complex with that of the Sema3A-PlexinA4-Nrp1 complex, based on either Nrp1-a1 or Nrp-a2, show severe clashes, indicating that Nrp1 cannot form these two complexes simultaneously (fig. S2B) (*27*).

The details of the contacts between the Nrp1 and VEGFR2 are well resolved in the cryo-EM map (Fig. 2, C and D, and fig. S1F). Nrp1-a1 and VEGFR2-Ig4 interact in a side-by-side fashion, with the outer surface of one of the two β sheets in Nrp1-a1 packing against one edge of the two β sheets in VEGFR2-Ig4 (Fig. 2C). The interface is dominated by hydrophobic interactions. At the center of the interface, Y38 in Nrp1-a1 makes packing interactions with F334 and H413 in VEGFR2-Ig4. Another hydrophobic interface patch is formed by Y44 and P45 from Nrp1-a1 and F331, A333, and L357 from VEGFR2-Ig4. There are also several interactions mediated by polar residues at the periphery of the interface, such as N35, N72, and R137 from Nrp1-a1 and S336, S340, and H413 from VEGFR2-Ig4.

Both the a1 and a2 domains in Nrp1 belong to the CUB (complement, Uegf, and BMP1) domain family, which is characterized by a Ca^2+^-binding site at one end of the β-sandwich (*27*). The calcium ion is coordinated by several negatively charged residues in the two interstrand loops. This site in CUB domains often mediates protein-protein interactions by binding to a lysine residue from the binding partners, as exemplified by the interactions of Nrp1-a1 and Nrp-a2 with plexin and semaphorin, respectively (*27*, *37*). The Ca^2+^-binding site in Nrp1-a1 is not involved in the interaction with VEGFR2-Ig4. In contrast, Nrp1-a2 engages VEGFR2-Ig4 in an orthogonal mode, placing the Ca^2+^-binding site at the center of the binding interface (Fig. 2D). K410 of VEGFR2-Ig4 interacts with E195 and D250 in the Ca^2+^-binding site in Nrp1-a1. This interaction is surrounded by additional contacts provided by residues from both proteins, including Y208, A252, and I253 from Nrp1-a2 and I401, M408, and S412 from VEGFR2-Ig4.

### Validation of the interface between VEGFR2 and Nrp1

The residues in the VEGFR2-Nrp1 interface are highly conserved in the two proteins from different species, supporting its physiological relevance (figs. S3A and S4A). To experimentally validate this interface, we measured the binding affinity between the ectodomains of VEGFR2 and Nrp1 with biolayer interferometry (BLI). We used a construct of Nrp1 containing the a1-a2-b1-b2 domains (residues 22 to 588), as the structure shows that the MAM domain and the flanking linkers are not involved in the interaction. The BLI results showed a weak interaction between VEGFR2 and Nrp1, with fast kinetics (*k*_on_ and *k*_off_ of 1.328 × 10^4^ M^−1^ s^−1^and 5.458 × 10^−1^ s^−1^, respectively) (Fig. 2E). The dissociation constant (*K*_D_) calculated from steady-state curve fitting is ~44.4 μM (Fig. 2E). The Y38A and Y38A/A252R mutations of Nrp1 at the binding interface markedly reduced the binding to VEGFR2, supporting the binding mode seen in the cryo-EM structure (Fig. 2F). Furthermore, we found that 5 mM EDTA in the buffer abolished the interaction between VEGFR2 and Nrp1, confirming the critical role of calcium ion at the binding interface (Fig. 2F).

Consistent with the weak affinity and fast off-rate of the interaction, VEGFR2 and Nrp1 failed to remain as a complex on SEC. We tested the interface mutations on SEC by taking advantage of the fact that VEGF_164_, VEGFR2, and Nrp1 together formed a stable complex. The results showed that the interface mutations in either Nrp1 (Y38A, S135R, A252R, and Y38A/A252R) or VEGFR2 (F331A, K410E, and S412N/M414T) reduced the formation of the VEGF_164_-VEGFR2-Nrp1 complex (figs. S3B and S4B). These results together with the cryo-EM structure strongly support the model in which Nrp1 (and likely Nrp2) directly interacts with both VEGFR2 and VEGF_164_, thereby promoting the dimerization and activation of VEGFR2. While the interaction between Nrp1 and VEGFR2 is weak in solution, it could be strengthened between full-length VEGFR2 and Nrp1 on the cell surface where both the proteins are confined to two-dimensional (2D) space and reach higher local concentrations.

### Structure of the heparin-bound cis tetrameric VEGF_164_-VEGFR2-Nrp1 complex

**Fig. 3.**
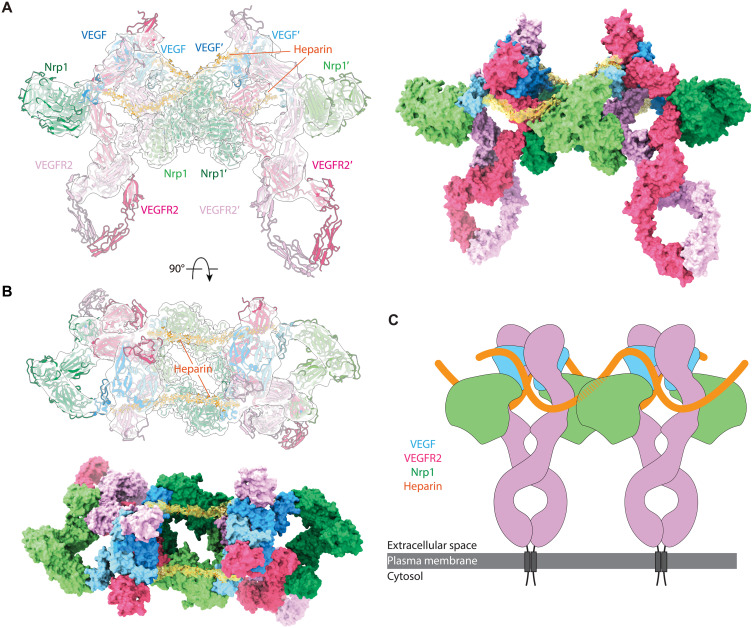
Cryo-EM structure of the cis tetrameric complex of VEGF_164_, VEGFR2, and Nrp1 induced by mucosal heparin. (**A**) Overall view of the cryo-EM map and the atomic model of the cis tetrameric complex. The left shows the cryo-EM density in semitransparent mode and the atomic model. The right shows the surface representation of the model. (**B**) Cryo-EM map and atomic model of the complex in a view orthogonal to that in (A). (**C**) Schematic model of the cis tetrameric complex.

Next, we sought to understand how HSPG may enhance the formation of the VEGF_164_-VEGFR2-Nrp1 complex. Adding mucosal heparin to the mixture of VEGF_164_, VEGFR2 and Nrp1 led to a sample that appeared much larger than the dimeric complex described above based on both SEC and cryo-EM images (figs. S5, A and B, and S6A). We solved the cryo-EM structure of the heparin-bound complex to an overall resolution of 3.3 Å (Fig. 3A and figs. S6 and S7). Notably, this complex shows a 4:4:4:2 stoichiometry, containing two copies of the dimeric protein complex and two long heparin chains (Fig. 3). The two dimeric complexes in this structure are very similar to the protein complex without heparin bound, with a root mean square deviation (RMSD) of the aligned Cα atoms of 1.2 Å. Therefore, it appears that heparin ties the two complexes together but does not induce substantial conformational changes in them. The two dimeric complexes are oriented roughly in the same direction, with their principal axes related by a ~58° angle, suggesting that this tetramer could form on the cell surface (fig. S8). We therefore refer to this complex as the cis tetrameric complex.

**Fig. 4.**
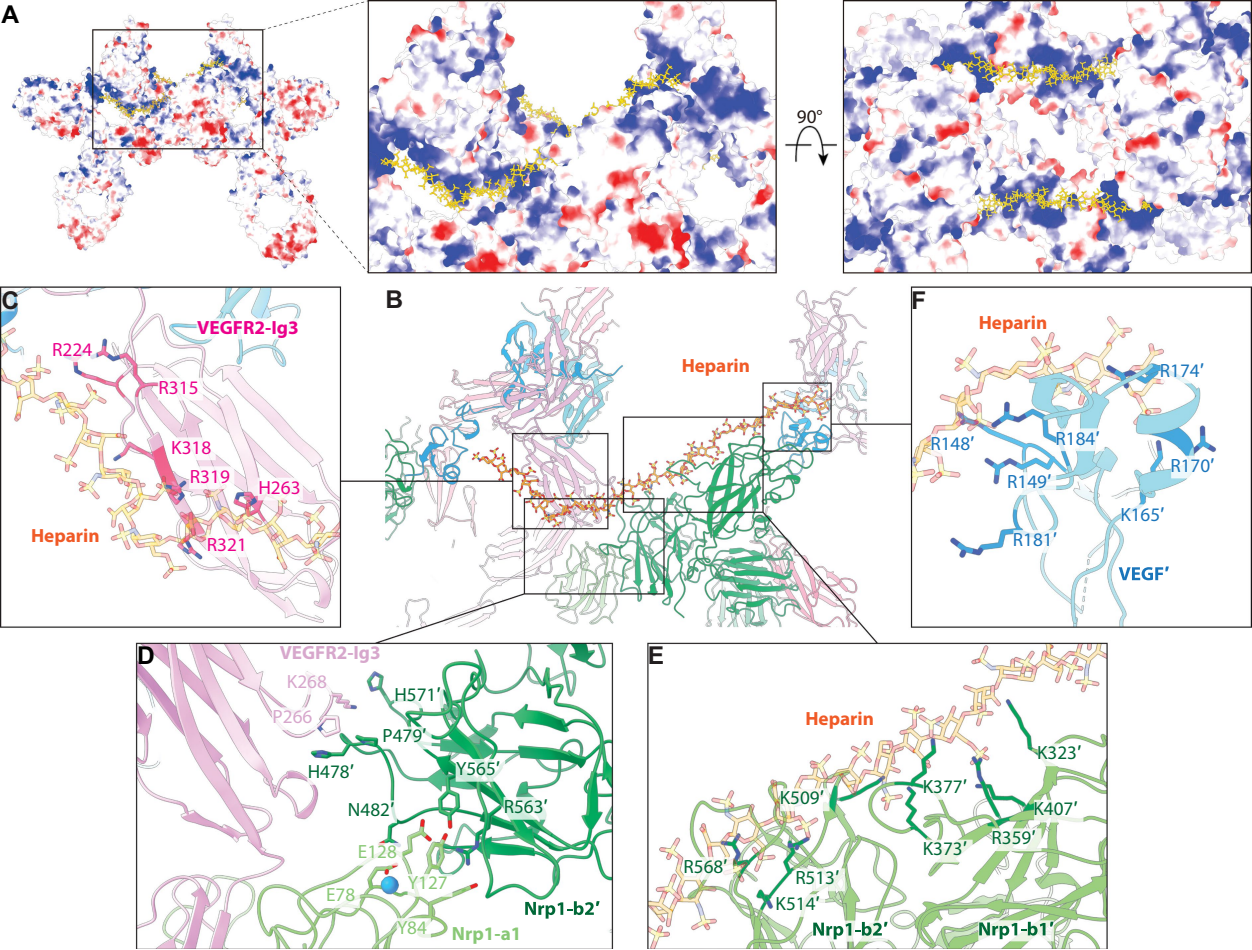
Interactions between the VEGF_164_-VEGFR2-Nrp1 complex and mucosal heparin. (**A**) Surface electrostatic potential of the cis tetrameric complex of VEGF_164_-VEGFR2-Nrp1 induced by mucosal heparin. Heparin molecules (yellow) bind the positively charged surface (blue) formed by VEGF_164_, VEGFR2, and Nrp1. (**B**) Overview of the interfaces between heparin and the protein complex. (**C**) Detailed view of the interface between heparin and VEGFR2-Ig3. (**D**) Detailed view of the interface between VEGFR2-Ig3 and Nrp1-a1 from the first dimeric complex and Nrp1′-b2 (Nrp1′ denotes Nrp1 from the second dimeric complex). (**E**) Detailed view of the interface between heparin and Nrp1′-b1-b2. (**F**) Detailed view of the interface between heparin and VEGF′ (VEGF′ denotes VEGF from the second dimeric complex).

The two dimeric complexes engage each other in a shoulder-to-shoulder configuration (Fig. 3). The b2 domain of one Nrp1 protomer from one dimeric complex makes contacts with both VEGFR2-Ig3 and Nrp1-a1 from the other dimeric complex (Figs. 3 and 4). The same interactions are formed in a reciprocal manner on the opposite side of the twofold symmetry axis. In principle, this shoulder-to-shoulder packing between the dimeric complexes is open-ended and could propagate to form higher-order oligomers. However, such longer oligomeric chains are unlikely to form on the same cell surface, as the fourth dimeric complex would point in the direction opposite to the first one given the 58° rotation between consecutive protomers (fig. S8).

### Heparin induces the cis tetrameric by interacting with all the three proteins simultaneously

The fact that we only observed the dimeric complex in the absence of heparin suggested that heparin is critical for the formation of the cis tetrameric complex. The cryo-EM density showed that two long heparin chains, modeled as 14 disaccharide units of sulfated iduronic acid (IDS) and sulfated glucosamine (SGN), extend across the two dimeric complexes (Fig. 3). One heparin chain starts at one side of the HBD of VEGF_164_ in the first dimeric complex and ends at the other side of the HBD of VEGF_164_ in the second dimeric complex. In the middle, it runs across the surfaces of VEGFR-Ig4, Nrp1-b2, and Nrp1-b1 sequentially (Fig. 4, A and B). The two heparin chains therefore promote the formation of the cis tetrameric complex by acting as molecular tethers to tie the two dimeric complexes together. The shoulder-to-shoulder configuration is stabilized by both heparin and protein-protein contacts (Figs. 3 and 4, B to F).

Because of the relatively poor density for heparin, the atomic details of its interactions with the proteins could not be resolved. Nevertheless, the structure reveals the parts in each protein that participate in heparin binding (fig. S7B). As expected, heparin interacts with the well-characterized, highly positively charged HBD in VEGF_164_, including R148, R149, K165, R170, R174, R181, and R184 (Fig. 4F) (*14*, *38*, *39*). Likewise, Npr1 uses the positively charged surface of the b1-b2 domains, formed by residues K323, R359, K373, K377, K407, K509, R513, K514, and R568, to bind heparin as reported by previous studies (Fig. 4E) (*28*, *30*). The heparin-binding site on VEGFR2 was largely unknown (*32*, *33*). Our structure shows that one side of the VEGFR2-Ig4 contains several positively charged residues, including R224, H263, R315, K318, R319, and R321, which mediate the interaction with heparin (Fig. 4C). VEGF_164_, VEGFR2, and Nrp1 together form a continuous positively charged surface to which the long heparin chain sticks, evident in colored rendering of the surface-based electrostatic potential (Fig. 4A). This binding mode therefore underlies the synergistic effect of the three proteins in interacting with heparin as reported previously (*33*).

### Heparin and Nrp1 together promote the activation of VEGFR2

**Fig. 5.**
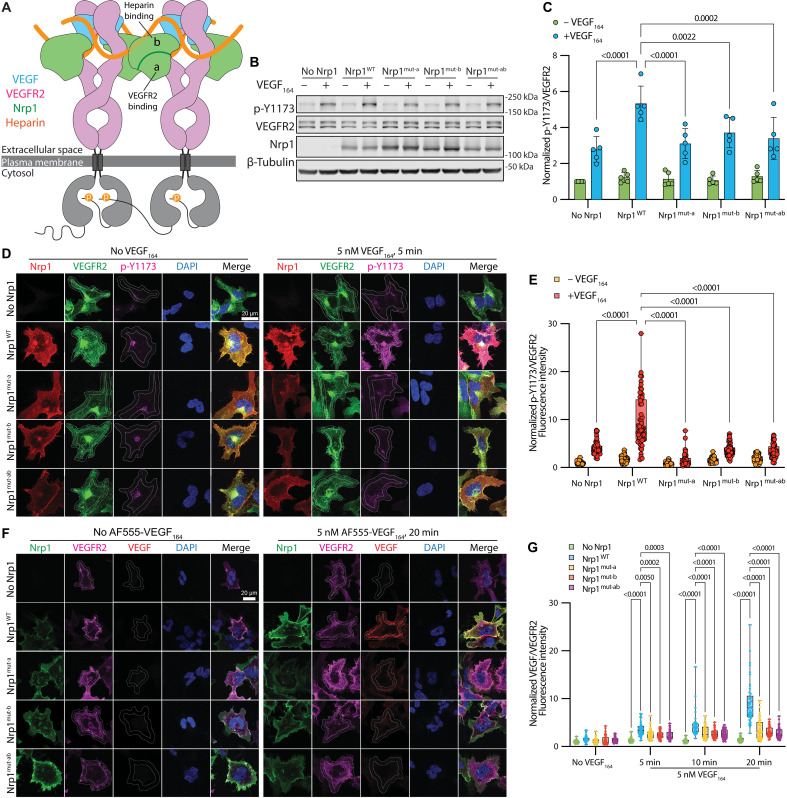
Enhancement of VEGF-induced VEGFR2 activation by Nrp1 and heparin in cells. (**A**) Schematic of heparin-induced cis tetrameric complex of VEGF, VEGFR2, and Nrp1 that promotes autophosphorylation of VEGFR2. (**B**) Western blot of VEGFR2 autophosphorylation in cells. HEK293T cells stably expressing VEGFR2 and the indicated Nrp1 constructs were stimulated with 5 nM VEGF_164_ for 5 min. Coexpression of wild-type Nrp1 increased the VEGF-stimulated VEGFR2 phosphorylation, which was reduced by mutating the binding interfaces for VEGFR2 and heparin on Nrp1. (**C**) Quantification of the result in (B). The values represent the ratios of the phosphorylated VEGFR2-Y1173 to total VEGFR2. Data shown are representatives of five biological repeats, normalized to the condition in the absence of both Nrp1 expression and VEGF stimulation. (**D**) Analyses of VEGF-stimulated VEGFR2-Y1173 phosphorylation by fluorescence microscopy. HEK293T cells stably expressing VEGFR2 alone or coexpressed with the indicated Nrp1 constructs were stimulated with VEGF_164_ and subjected to immunostaining. (**E**) Quantification of the intensity ratio of p-Y1173 to VEGFR2 of cells in (D). (**F**) Analyses of VEGF binding to cells expressing VEGFR2 alone or with the indicated Nrp1 constructs. Cells were stimulated with 5 nM AF555-labeled VEGF_164_ and subjected to immunostaining. (**G**) Quantification of the intensity ratio of AF555-mVEGF_164_ to VEGFR2 of cells in (F). For (D) to (G), the peripheral area around the plasma membrane of the cell (between the two white lines) was set as the region of interest for quantification, ensuring the exclusion of VEGFR2 in the ER or Golgi that was not accessible to VEGF. Values were normalized to the baseline condition (no Nrp1 coexpression and no VEGF_164_ stimulation) and presented as mean ± SEM. The experiments were repeated three times. The number of analyzed cells was 60 and 45 in (E) and (G), respectively. The *P* values were calculated using two-tailed Student’s *t* test. DAPI, 4′,6-diamidino-2-phenylindole.

The cis tetrameric complex provides a mechanistic basis for the role of neuropilin and heparin in promoting the formation of the active dimer and higher-order clusters of VEGFR2 for enhanced signaling (Fig. 5A). To validate the heparin-binding mode, we designed several sets of mutations of heparin-binding residues in Nrp1, including K323A/K373A/K377A in Nrp1-b1 (referred to as Nrp1^mut-b1^), R513A/K514A/R586A in Nrp1-b2 (Nrp1^mut-b2^), and the combination of the two (Nrp1^mut-b^). As expected, these mutants did not affect the formation of the dimeric VEGF_164_-VEGFR2-Nrp1 complex in the absence of heparin (fig. S9, A and B). However, the complex formed by Nrp1^mut-b1^, Nrp1^mut-b2^, or Nrp1^mut-b^ in the presence of heparin eluted much later than the wild-type complex on SEC (fig. S9, C and D). These results support the notion that heparin is required for the high-order oligomerization of the VEGF_164_-VEGFR2-Nrp1 complex.

We next tested whether Nrp1 and heparin enhance the VEGF_164_-induced autophosphorylation of VEGFR2 in human embryonic kidney (HEK) 293T cells stably expressing full-length VEGFR2. The Western blot results showed that coexpression of Nrp1 in these cells led to increased autophosphorylation of Y1173, a major autophosphorylation site in VEGFR2 (Fig. 5, B and C). This effect of Nrp1 was diminished by mutation of the VEGFR2-binding site (Y38A/A252R, referred to as Nrp1^mut-a^), the heparin-binding sites (Nrp1^mut-b^), or both (Nrp1^mut-ab^). However, while statistically significant, the effects of the mutations appeared small. It is well recognized that the VEGF-stimulated phosphorylation of VEGFRs can be masked in this type of bulk assays by several factors, including nonresponsive VEGFR2 retained in the endoplasmic reticulum and Golgi apparatus, as well as constitutive autophosphorylation resulting from excessive overexpression (*40*). To overcome these issues, we used fluorescence microscopy to quantify the autophosphorylation of VEGFR2 expressed on the cell surface of individual HEK293T cells while excluding intracellular VEGFR2, following an approach similar to that described previously (*40*). The results showed that the VEGF-stimulated phosphorylation levels of Y1173 in VEGFR2 were significantly higher in cells coexpressing Nrp1 with VEGFR2 compared with those expressing VEGFR2 alone (Fig. 5, D and E). In contrast, the Nrp1 mutants (Nrp1^mut-a^, Nrp1^mut-b^, and Nrp1^mut-ab^) lost the ability to enhance VEGFR2 autophosphorylation. Last, we examined whether Nrp1 and heparin facilitate the binding of VEGF_164_ to VEGFR2 using the same set of cells. Consistent with the results of VEGFR2 autophosphorylation, wild-type Nrp1 showed strong effects on increasing the binding of fluorescence-labeled VEGF_164_ to the cell surface, which was diminished or abrogated by the Nrp1 mutations that disrupt the binding of either VEGFR2 or heparin (Fig. 5, F and G). These results together substantiate our structure-based model that neuropilin and heparin function cooperatively with VEGF to promote the clustering and activation of VEGFR2.

### Structure of the trans tetrameric VEGF_164_-VEGFR2-Nrp1 complex induced by short-chain heparin

**Fig. 6.**
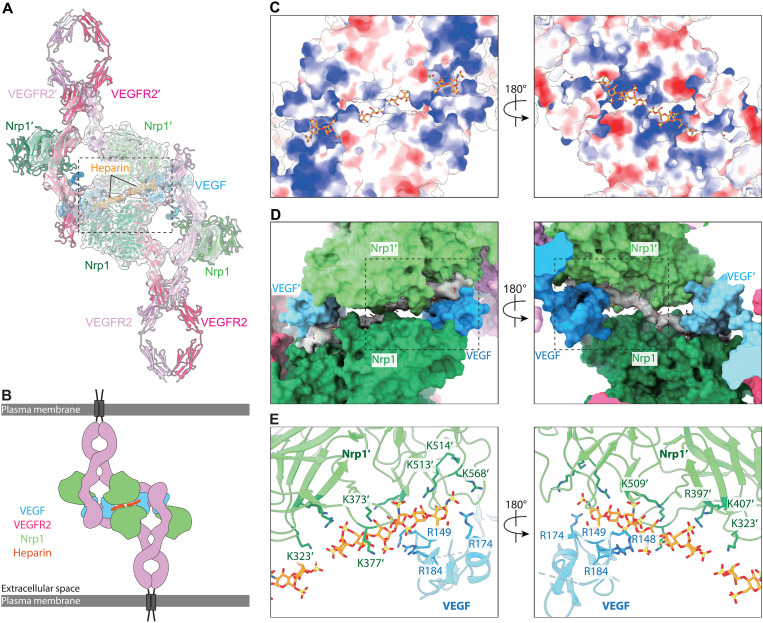
Cryo-EM structure of the trans tetrameric complex of VEGF_164_, VEGFR2, and Nrp1 induced by heparin dp12. (**A**) Overview of the cryo-EM map and atomic model of the trans tetrameric complex. (**B**) Structure-based model of heparin-induced trans tetrameric complex of VEGF, VEGFR2, and Nrp1 from two adjacent cells. (**C**) Surface electrostatic potential of heparin-binding site in the trans tetrameric complex. The part shown corresponds to the boxed region in (A). Red and blue indicate negative and positive electrostatic potentials, respectively. (**D**) Surface representation of an expanded view of the heparin-binding site. The density for heparin dp12 is shown in gray. (**E**) Detailed view of heparin dp12 binding interface formed by Nrp1 and VEGF_164_.

We also reconstituted heparin-bound VEGF_164_-VEGFR2-Nrp1 complex using heparin with an average chain length of 12 saccharide units [referred to as heparin degree of polymerization 12 (dp12)], much shorter than mucosal heparin (~50 saccharide units, dp50). The heparin dp12-bound complex appeared much larger than the dimeric protein complex based on SEC (fig. S5, C and D). Consistently, our cryo-EM structure of this complex at 3.6-Å resolution revealed a tetrameric assembly containing two dimeric complexes, each of which is similar to the protein-only dimeric complex (RMSD for the aligned Cα atoms of 2.2 Å) (Fig. 6 and fig. S10). However, the two dimers adopt an antiparallel configuration, distinct from the cis tetrameric complex described above (Fig. 3A). The two dimeric complexes are related by a 162° rotation, causing the C termini of the VEGRF2 molecules in the two dimeric complexes pointing in opposite directions. We therefore refer to this assembly as the trans tetrameric complex, as it is more likely to form by two dimeric complexes from opposing surfaces of two neighboring cells (Fig. 6, A and B).

The interaction between the two dimeric complexes is mediated by heparin dp12, the Nrp1-b1-b2, and HBDs of VEGF_164_ together (Fig. 6C). The Nrp1-b1-b2 and HBDs of VEGF_164_ on one side of the first dimeric complex are juxtaposed against its counterpart from the second dimeric complex. As a result, the heparin-binding surfaces of these domains together form a positively charged tunnel, with the twofold symmetry axis located at the middle (Fig. 6, C and D, and fig. S10G). On the basis of the density, we built two short heparin chains of 3 disaccharide units, each sitting on one half of the tunnel, although it is possible one longer chain with 6 or more disaccharide units occupies the entire tunnel. The residues in Nrp1 and VEGF_164_ involved in the interaction with heparin are mostly the same as those in the cis tetrameric complex (Fig. 6E). However, VEGFR2 is not involved in the interaction in this case, leading to a much smaller heparin-binding surface. It appears that the shorter chain length of heparin dp12 can only support the formation of the trans tetrameric complex, whereas longer chain mucosal heparin favors the cis tetrameric complex.

Our SEC results showed that mutating the heparin-binding sites in Nrp1 reduced the size of the high-order complex of VEGF_164_-VEGFR2-Nrp1 induced by heparin dp12, supporting the trans tetrameric complex model (fig. S9, E and F). The functional role of this trans tetrameric complex is not certain at this point, although it could represent the trans complexes of VEGFR formed between opposing surfaces of two neighboring cells. These trans VEGFR complexes have been proposed to prolong and enhance signaling by trapping the active receptor at the cell surface (*41*–*43*). Nrp1 uses largely the same interfaces for binding VEGFR2 and heparin in both the cis and trans tetrameric complexes, and therefore the Nrp1 mutations described above can disrupt both assemblies. However, our microscopic assay showed that Nrp1-mediated enhancement of VEGFR2 phosphorylation occurred in areas of cell membrane with or without cell-cell contacts, indicating that the effect does not depend on the trans interactions between adjacent cells (Fig. 5D). Under these experimental conditions, the mutations therefore likely reduced VEGFR2 phosphorylation by impairing the cis tetrameric complex, although a contribution from the trans complex cannot be excluded.

## DISCUSSION

Given its critical role in angiogenesis, the VEGF_164_/VEGFR2 signaling axis is subjected to tight regulation by both regulatory proteins and heparin. In this study, our cryo-EM structures reveal the molecular details of how neuropilin and heparin together promote the active dimer and high-order oligomers of VEGFR2 without inducing any substantial conformational change within the individual proteins. The binding interfaces observed in our structures could be the basis for developing new therapeutics for diseases such as cancer and macular degeneration. In this regard, it is interesting to note that the Ig4 in VEGFR2 where Nrp1 binds has been targeted by designed ankyrin repeat proteins (DARPin), which act as allosteric inhibitors (*44*). One of the mechanisms by which the DARPin proteins inhibit VEGFR2 signaling may be preventing Nrp1 binding, as their binding sites partially overlap (fig. S2E).

A superimposition of our structure with the structures of VEGFR1 [Protein Data Bank (PDB) ID: 5T89] and VEGFR3 (PDB ID: 4BSJ) shows that both VEGFR1 and VEGFR3 have an N-glycosylation site in Ig4, on N417 and N411 respectively, corresponding to S412 in VEGFR2-Ig4 that is located at the center of the binding surface with Nrp1-a2 (fig. S3, A and C). These N-glycan groups in VEGFR1 and VEGFR3 would clash with Nrp1-a2 and thereby prevent its binding. In addition, other residues in VEGFR2-Ig4 involved in Nrp1 binding are not conserved in VEGFR1 or VEGFR3 (fig. S3A). These analyses together suggest that the interaction with Nrp1-a1-a2 is a feature unique to the Ig4 domain of VEGFR2. Consistently, our SEC data suggested that VEGFR1 failed to form a stable 2:2:2 dimeric complex with both VEGF_164_ and Nrp1 (fig. S3D). However, several studies have suggested that VEGFR1 interacts with neuropilin (*16*, *17*), which may be mediated by an interface different from the VEGFR2/Nrp1 interface seen in our cryo-EM structure.

The cis tetrameric complex induced by mucosal heparin suggests a mechanism for clustering of VEGFR2 on the cell surface, which could increase signaling by both stabilizing the active kinase dimer and promoting local condensates that concentrate signaling components. Micrographs of our cryo-EM sample contained filament-like particles that appeared to be composed of multiple copies of the tetrameric complex reminiscent of daisy chains, consistent with the early elution volume of the mucosal heparin-bound VEGF_164_-VEGFR2-Nrp1 sample in the presence of heparin (figs. S5A and S6A). Open-ended propagation of the interdimer interaction as seen in our cryo-EM structure may account for some of the larger complexes, although they cannot form on the same cell surface due to the ~58° rotation between consecutive dimeric units (fig. S8). On the other hand, larger complexes could arise through simple tethering of multiple tetrameric complexes by long heparin chains. They could form on the cell surface as the relative orientation of the tetrameric units is not constrained. It is possible that both the ordered cis tetrameric complex and the flexibly tethered oligomers coexist in vivo, which together underlie VEGFR2 clustering on the cell surface as shown by previous studies (*45*, *46*).

As mentioned above, some studies have suggested that the formation of trans complexes of VEGFRs by components from opposing surfaces of two cells could enhance signaling by trapping the active receptor at the cell surface (*41*–*43*). Conversely, endocytic trafficking and signaling endocytic vesicles have been proposed to be an important aspect of VEGFR2 signaling, and prolonged cell surface retention by trans complexes may instead attenuate signal (*26*, *41*, *47*–*49*). Our trans-tetrameric complex suggests a mechanism for the formation of trans interactions among the three proteins and heparin. In addition to this structurally defined mechanism, trans complexes VEGFR could form through the tethering effects of different components from neighboring cells (*48*). For example, our structures suggest that long-chain HSPG from one cell could bind the VEGF_164_-VEGFR2-Nrp1 complex on another cell in trans. Similarly, the long and flexible region following the b2 domain in neuropilin could also allow it to form trans dimeric complexes with VEGF_164_-VEGFR2. Moreover, a previous study has shown that the linker between the b2 and MAM domains in Nrp1 contains a site of modification by glycosaminoglycan, which could contribute to either the cis or trans tetrameric complex formation (*50*).

HSPGs on the cell surface or in the extracellular matrix have been reported to span a wide range of chain lengths (20 to 200 disaccharide units) and exhibit highly diverse sulfation patterns (*51*). A limitation of our study is that it does not address how sulfation patterns may regulate the binding of heparan sulfate to the VEGF-VEGFR2-Nrp1 complex, due to the relative low resolution of the cryo-EM density for the heparin molecules. On the basis of the comparison of the two heparin-bound structures, the long saccharide chain lengths in vivo are likely to favor formation of the cis tetrameric complex, thereby promoting VEGFR2 clustering and signaling. However, the trans tetrameric complex may form under certain conditions, as other factors, such as cell-cell contacts and protein expression levels could shift the balance between the two.

**Fig. 7.**
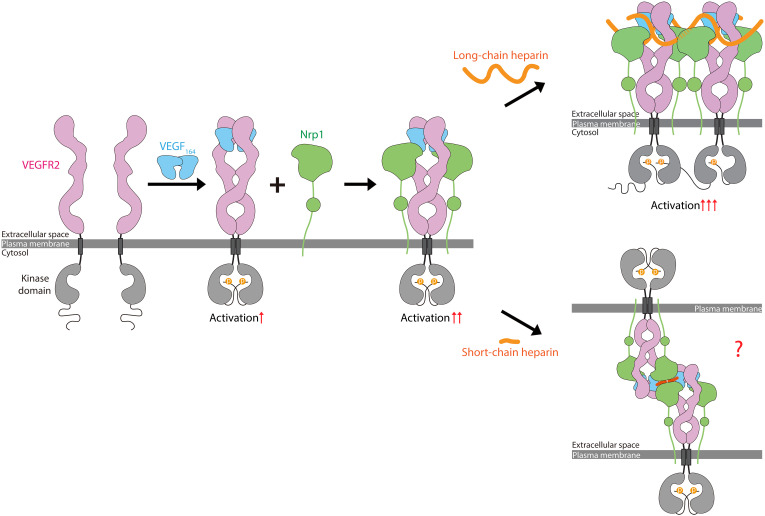
Model of cooperative activation of VEGFR2 by VEGF, neuropilin, and heparin. Our experimental evidence suggests that the cis tetrameric complex can enhance VEGFR2 clustering and signaling. The existence of the trans tetrameric complex between opposing cells is less certain, indicated by the question mark.

The findings of our study establish a structural framework for further studies that aim to understand the complexity and functional roles of the high-order oligomers of VEGFR2 promoted by VEGF, neuropilin, and heparin/heparan sulfate together in either cis or trans (Fig. 7). More broadly, heparin and HSPG are known to bind and regulate signaling of many cell surface receptors and their ligands, but the mechanisms for these interactions are understood only for a limited subset. A classic example is the fibroblast growth factor receptor, where heparin/heparan sulfate directly binds between the two receptor molecules and facilitates their dimerization (*52*). Two recent studies have shown that heparin/heparan sulfate binds the axon guidance molecule semaphorin and induces its oligomerization, which plays a regulatory role in the activation of the plexin receptor (*53*, *54*). A distinct feature in our structures is that heparin promotes the high-order oligomerization by interacting simultaneously with the ligand, receptor, and coreceptor. Future structural studies will likely uncover an even broader range of mechanisms underlying heparin-mediated regulation of receptor signaling.

## MATERIALS AND METHODS

### Protein expression and purification

Extracellular regions of mouse VEGFR2 (residues 20 to 760), Nrp1 (residues 22 to 855), and Nrp1 (residues 22 to 588) were cloned into a modified pEZT-BM vector encoding an N-terminal signal peptide from chicken receptor–type tyrosine-protein phosphatase S and a C-terminal 8xHis-tag (*55*). Mouse VEGF_164_ was cloned into pEZT-BM with an N-terminal 8xHis-tag. HEK293F cells were maintained in 400 ml of Freestyle 293 Expression Medium (Gibco) and grown to 2.0 × 10^6^ cells/ml. Cells were transiently transfected with 0.4 mg of plasmid mixed with 1.2 mg of polyethyleneimine (Polysciences Inc.). Cells were fed with 8 mM sodium butyrate at 18 to 22 hours after transfection. Media were collected 6 days after transfection by 15-min centrifugation at 3500 rpm and incubated with 1 ml of Ni Sepharose beads at 4°C for 2 hours. Beads were washed six times with 10 ml of buffer [20 mM tris-HCl (pH 8.0), 500 mM NaCl, and 5% glycerol] containing 20 and 40 mM imidazole, respectively. Proteins were eluted using elution buffer [20 mM tris-HCl (pH 8.0), 300 mM NaCl, 5% glycerol, and 500 mM imidazole]. Eluted proteins were concentrated and applied to SEC [Superose 6 (Cytiva) for VEGFR2 and Nrp1 and Superdex 200 (Cytiva) for VEGF_164_ in 20 mM Hepes (pH 7.0), 150 mM NaCl, and 1 mM CaCl_2_]. Fractions containing the target proteins based on SDS–polyacrylamide gel electrophoresis (SDS-PAGE) analyses were collected, concentrated, snap-frozen in liquid nitrogen, and stored at −80°C before use.

### Reconstitution of the VEGF_164_-VEGFR2-Nrp1 with or without heparin

The VEGF_164_-VEGFR2-Nrp1 complex was reconstituted by mixing 25 μM of purified VEGF_164_, VEGFR2 (20 to 760), and Nrp1 (22 to 855) in a buffer containing 20 mM Hepes (pH 7.0), 150 mM NaCl, and 1 mM CaCl_2_. After 2-hour incubation, the sample was subjected to SEC on a Superose 6 column. Fractions were analyzed by SDS-PAGE, and peak fractions containing the protein complex were pooled and concentrated. For preparing the heparin-bound VEGF_164_-VEGFR2-Nrp1, the protein complex at 12.5 μM was mixed with 2.5 μM mucosal heparin (Sigma-Aldrich) or 12.5 μM heparin oligosaccharide dp12 (Iduron, HO12). SEC with the Superose 6 column showed that heparin binding led to earlier elution of the complex, suggesting increased size. Peak fractions eluted at around 11 ml were pooled and concentrated. Binding analysis of protein mutants in heparin-bound VEGF_164_-VEGFR2-Nrp1 oligomer complex formation was carried out under the same condition.

### Cryo-EM sample preparation, data collection, and image processing

The VEGF_164_-VEGFR2-Nrp1 complex, the complex bound to mucosal heparin, and that bound to heparin dp12 at 0.4 mg/ml were used for cryo-EM grid preparation. Samples were applied to a glow-discharged Quantifoil R1.2/1.3 300-mesh gold holey carbon grid (Quantifoil, Micro Tools GmbH, Germany). Grids were blotted under 100% humidity at 4°C and plunged into liquid ethane with a Mark IV Vitrobot (FEI). Micrographs of the VEGF_164_-VEGFR2-Nrp1 complex and the protein complex with heparin dp12 bound were collected on a Titan Krios microscope (FEI) equipped with a K3 summit direct electron detector (Gatan). Micrographs of the complex with mucosal heparin bound were collected on a Titan Krios microscope (FEI) equipped with a Falcon 4 detector (Thermo Fisher Scientific). Both the microscopies were controlled using the SerialEM software version 4 (*56*). Parameters of data collection are summarized in table S1.

Motion correction and dose weighting of the micrographs were carried out using RELION (version 5) and Motioncor2 programs for data collected with the Falcon 4 and Gatan K3 detectors, respectively (*57*, *58*). CTFFIND4 was used for Contrast Transfer Function (CTF) correction (*59*). For the protein-only complex and the complex with heparin dp12 bound, particles picked by templated-based particle picking using RELION5 were used for the subsequent processing steps. For the complex with mucosal heparin bound, particles from templated-based picking and those from artificial intelligence-assisted picking with Cryolo v1.9 (*60*) were combined. The following data processing steps, including 2D classification, initial model generation, 3D classification, 3D refinement with the BLUSH algorithm, CTF refinement, Bayesian polishing, and postprocessing, were all carried out in RELION 5. The C2 symmetry was imposed for all the three structures. The Fourier shell correlation (FSC) of the two half maps with the threshold of 0.143 was used for estimating the resolution of the cryo-EM maps. Local resolution of the maps was calculated using the method implemented in RELION. The data processing procedures are summarized in figs. S1, S6, and S10.

### Model building, refinement, and validation

Model building of the dimeric VEGF_164_-VEGFR2-Nrp1 complex was initiated by docking the AlphaFold models of mouse VEGF (AF-Q00731-3-F1-v2), VEGFR2 (AF-P35918-F1-v2), and Nrp1 (AF-P97333-F1-v2) into the cryo-EM map in ChimeraX 1.8 (*61*). The first Ig-like domain of VEGFR2 and the region C-terminal to the b2 domain of Nrp1 were not included in the atomic model due to lack of density. The N-terminal domain in VEGF_164_, VEGFR2 Ig2 to lg5, and Nrp1 a1, a2, b1, and b2 were well resolved (Fig. 1 and fig. S1). Densities for Ig-like 6 and 7 in VEGFR2 and the heparin-binding domain of VEGF_164_ were weak, which allowed the domains to be placed but details of residues in these domains are not resolved. Calcium ions bound to the a1 and a2 domains of Nrp1 and N-glycans were added into the models using the ISOLDE tool (version 1.8) in ChimeraX (*62*).

The cis tetrameric complex with mucosal heparin bound and the trans tetrameric complex with heparin dp12 bound were constructed by docking two copies of the dimeric complex into the cryo-EM maps. Extra elongated densities near the known heparin-binding sites in Nrp1 and VEGF_164_ were evident once the proteins were placed into the maps. Heparin dp12 and mucosal heparin are heterogeneous mixtures of heparin chains with average lengths of 12 and 50 of saccharide units, respectively. The most common forms of heparin contain disaccharide units of 2-O-IDS and 6-O-sulfated, *N*-SGN (*63*). On the basis of the densities, we built two copies of heparin chains containing 14 IDS-SGN units into the mucosal heparin-bound tetrameric complexes. Because of the relatively weak and low-resolution density, the orientation of the heparin chains could not be assigned unambiguously. We arbitrarily choose one orientation to build the heparin chains, although the oppose orientation was also possible. These ambiguities prevent us from discerning the interactions between the protein and the heparin chains in detail, but the overall binding mode is clear. The heparin density in the trans tetrameric complex with heparin dp12 was much shorter, which were fit with two heparin chains of 3 IDS-SGN units. The two heparin chains meet at the twofold symmetry axis in the structure. It is possible that one longer heparin chain occupies both the two binding sites, given the repetitive nature of heparin and the fact that the interactions between heparin and proteins are dominated by electrostatic interactions that do not rely on precise individual specific contacts.

The models were manually adjusted and refined with molecular dynamics–based elastic refinement using ISOLDE. The first Ig-like domain of VEGFR2 and the region after the b2 domain of Nrp1 (the MAM domain and the flexible linkers flanking the MAM domain) were not included in the atomic models of the three structures due to lack of density. The models were subjected to further positional and B-factor refinement using the real-space refinement module in Phenix 1.18 with twofold symmetry, Ramachandran, and secondary structure restraints (*64*). Model quality was assessed using MolProbity as a part of the Phenix validation tool set (*65*). The FSC between the models and cryo-EM maps was calculated in Phenix. Model statistics are summarized in table S1. Structural figures were rendered in ChimeraX 1.8.

### Biolayer interferometry

The direct interaction between the ectodomain of VEGFR2 (residues 20 to 760) and Nrp1 (residues 22 to 588) was measured using BLI on an Octet R2 instrument (Sartorius). BLI experiments were carried out at 30°C with agitation set to 1000 rpm. VEGFR2 was biotinylated using the EZ-Link NHS-PEG4 Biotinylation Kit according to the manufacturer’s instruction (Thermo Fisher Scientific). Biotin-labeled VEGFR2 was purified through SEC to remove excess biotin. The protein was diluted in the assay buffer [20 mM Hepes (pH 7.5), 150 mM NaCl, 1 mM CaCl_2_, 0.1% bovine serum albumin (BSA), and 0.02% Tween 20] to 8 μg/ml and immobilized on a streptavidin biosensor (Sartorius). Association measurements were performed by dipping sensors into Nrp1 solutions at concentrations in the range of 50, 16.67, 5.56, 1.85, and 0.62 μM for 180 s. Dissociation was monitored by transferring the sensors to the assay buffer for 240 s. All analyte solutions were run in parallel with reference sensors to correct for nonspecific binding and bulk refractive index changes. Data analysis was performed using Octet Data Analysis software (Sartorius). Steady-state responses (*R*_eq_) were extracted from association curves for each Nrp1 concentration. A 1:1 binding model with steady-state analysis was applied.

### Stable cell line establishment

Lentiviral particles encoding Myc-tagged mouse VEGFR2 or hemagglutinin (HA)–tagged mouse Nrp1 were produced in HEK293T cells. Briefly, HEK293T cells were seeded in six-well plates and transfected with the pLVX-Puro-myc-mVEGFR2 or pLVX-Bsd-HA-mNrp1 plasmids together with packaging the plasmids psPAX2 (Addgene) and pMD2.G (Addgene) at a ratio of 5:3:3 using Lipofectamine 3000 according to the manufacturer’s protocol (Invitrogen). Viral supernatants were collected twice at 48 and 72 hours after transfection, cleared by centrifugation (300*g*, 5 min), and filtered through a 0.45-μm filter.

For transfection, HEK293T cells were seeded in six-well plates and incubated with lentiviral supernatants containing polybrene (8 μg/ml) for 72 hours. The medium was replaced with fresh complete Dulbecco’s modified Eagle’s medium and selection antibiotics, puromycin (2 μg/ml) for VEGFR2, and blasticidin (10 μg/ml) for Nrp1. Selection was continued for 5 to 7 days until nontransduced control cells were eliminated. Single-cell clones were isolated by fluorescence-activated cell sorting based on surface staining with a phycoerythrin-conjugated anti-Myc-tag antibody (1:100; BioLegend) and an Alexa Fluor 488–conjugated anti-HA tag antibody (1:100; BioLegend). Cells were stained on ice and washed twice with ice-cold presorting buffer (BD). Double-positive cells were sorted (100-μm nozzle) into 96-well plates containing conditioned medium. Clones were expanded, screened for receptor expression by immunoblotting, and maintained under continuous selection [puromycin (2 μg/ml) or blasticidin (10 μg/ml)] to ensure stable expression of VEGFR2 and Nrp1.

### Western blot

HEK293T cells stably expressing VEGFR2 and Nrp1 were seeded in six-well plates at 1 × 10^6^ cells per well. Twenty-four hours later, cells were serum starved for 6 hours and then stimulated with 5 nM VEGF_164_ for 5 min. Cells were washed with ice-cold PBS two times and lysed in radioimmunoprecipitation assay buffer supplemented with Halt Protease and Phosphatase Inhibitor Cocktail (Thermo Fisher Scientific) on ice for 20 min. After centrifugation, protein concentrations in supernatants were quantified using the bicinchoninic acid (BCA) protein assay (Thermo Fisher Scientific). The 4× SDS-PAGE loading buffer was added to lysates before heating at 70°C for 10 min. Ten micrograms per sample was resolved with 8% SDS-PAGE gels and transferred to 0.45-μm Nitro Cellulose membranes (Bio-Rad) by wet transfer at 200 mA for 2 hours at 4°C. Membranes were blocked in Tris-Buffered Saline with Tween 20 (TBST) with 5% BSA for 1 hour at room temperature and immunoblotted with the following primary antibodies overnight at 4°C: mouse anti-Myc (1:2000; Cell Signaling Technology), rabbit anti–phospho-Y1175 VEGFR2 (1:2000; Cell Signaling Technology), rabbit anti–neuropilin 1 (1:2000; Cell Signaling Technology), and mouse anti–β-tubulin (1:3000; Cell Signaling Technology). Membranes were washed with TBST, followed by secondary antibody incubation (anti-mouse IgG Dylight 680 conjugate, 1:15,000; anti-rabbit IgG Dylight 800 conjugate, 1:30,000) for 1 hour at room temperature. Blots were visualized using an Odyssey CLx Imaging System (LI-COR). Band intensities were quantified with Image Studio software (LI-COR). Phospho-Y1173 VEGFR2 signals were normalized to total VEGFR2 signals and reported as mean ± SEM from five biological replicates.

### Immunofluorescence

Cells were seeded onto poly-d-Lysine (Gibco) pretreated microscope coverslips (Alkali Scientific) placed inside 24-well cell culture plates at 4 × 10^4^ cells per well. Twenty-four hours after seeding, cells were serum starved for 6 hours, followed by stimulation with 5 nM Alexa Flour 555–labeled VEGF_164_ for indicated time or with 5 nM unlabeled VEGF_164_ for 5 min. Cells were washed twice with ice-cold PBS, fixed with 4% paraformaldehyde for 15 min at room temperature, and then permeabilized with 0.1% Triton X-100. Coverslips were blocked in 1× Dulbecco’s Phosphate-Buffered Saline (DPBS) containing 1% BSA and 0.05% Tween 20 for 1 hour at room temperature. For VEGFR2 phosphorylation assay, cells were probed with mouse anti-myc (1:1000; Cell Signaling Technology), chicken anti-HA (1:1000; Novus), rabbit anti–phospho-Y1175 VEGFR2 (1:1000; Cell Signaling Technology), followed with Alexa Fluor 488–conjugated goat anti-mouse secondary antibody (1:1000; Invitrogen), Alexa Fluor 555–conjugated goat anti-chicken secondary antibody (1:1000; Invitrogen), and Alexa Fluor 647–conjugated goat anti-rabbit secondary antibody (1:1000; Invitrogen). For VEGF localization assay, cells were probed with rabbit anti-HA (1:1000; Cell Signaling Technology), mouse anti-myc (1:1000; Cell Signaling Technology), followed with Alexa Fluor 488–conjugated goat anti-rabbit secondary antibody (1:1000; Invitrogen) and Alexa Fluor 647–conjugated goat anti-mouse secondary antibody (1:1000; Invitrogen). Nuclei were stained with 4′,6-diamidino-2-phenylindole before mounting coverslips to glass slides with Aqua/Poly Mount mounting medium (Polysciences Inc.). Images were taken on a Zeiss LSM780 inverted confocal laser scanning microscope with a 40× oil objective.

To quantify VEGF-simulated phosphorylation of VEGFR2, the area around the edge of the cell was chosen as the region of interest to exclude VEGFR2 retained in the ER or Golgi using the software Napari (https://napari.org/stable/). The level of phosphorylation was measured by calculating the ratio between the fluorescence signal of Y1173-phosphorylated VEGFR2 and that of VEGFR2 protein in the same area. The quantification of binding of labeled VEGF_164_ to the cell surface was carried out in a similar manner. Quantification data from three independent experiments were pooled for calculating the statistical significance. The images were rendered with the QuickFigures plugin in ImageJ (*66*, *67*).
