# Supplementary material for: Mechanisms of VEGFR2 activation by VEGF, neuropilin, and heparin
Source: Sci Adv. 2026 Jul 15;12(29):eaeg6323. doi: 10.1126/sciadv.aeg6323 (PMC13371918; doi:10.1126/sciadv.aeg6323)
Supplement: Supplementary file 1 — Figs. S1 to S10 Table S1 [file sciadv.aeg6323_sm.pdf]

Science Advances
AAAS

Supplementary Materials for

Mechanisms of VEGFR2 activation by VEGF, neuropilin, and heparin

Lianqi Chen *et al.*

Corresponding author: Xiao-chen Bai, xiaochen.bai@utsouthwestern.edu;
Xuewu Zhang, xuewu.zhang@utsouthwestern.edu

Sci. Adv. **12**, eaeg6323 (2026)
DOI: 10.1126/sciadv.aeg6323

This PDF file includes:

Figs. S1 to S10
Table S1

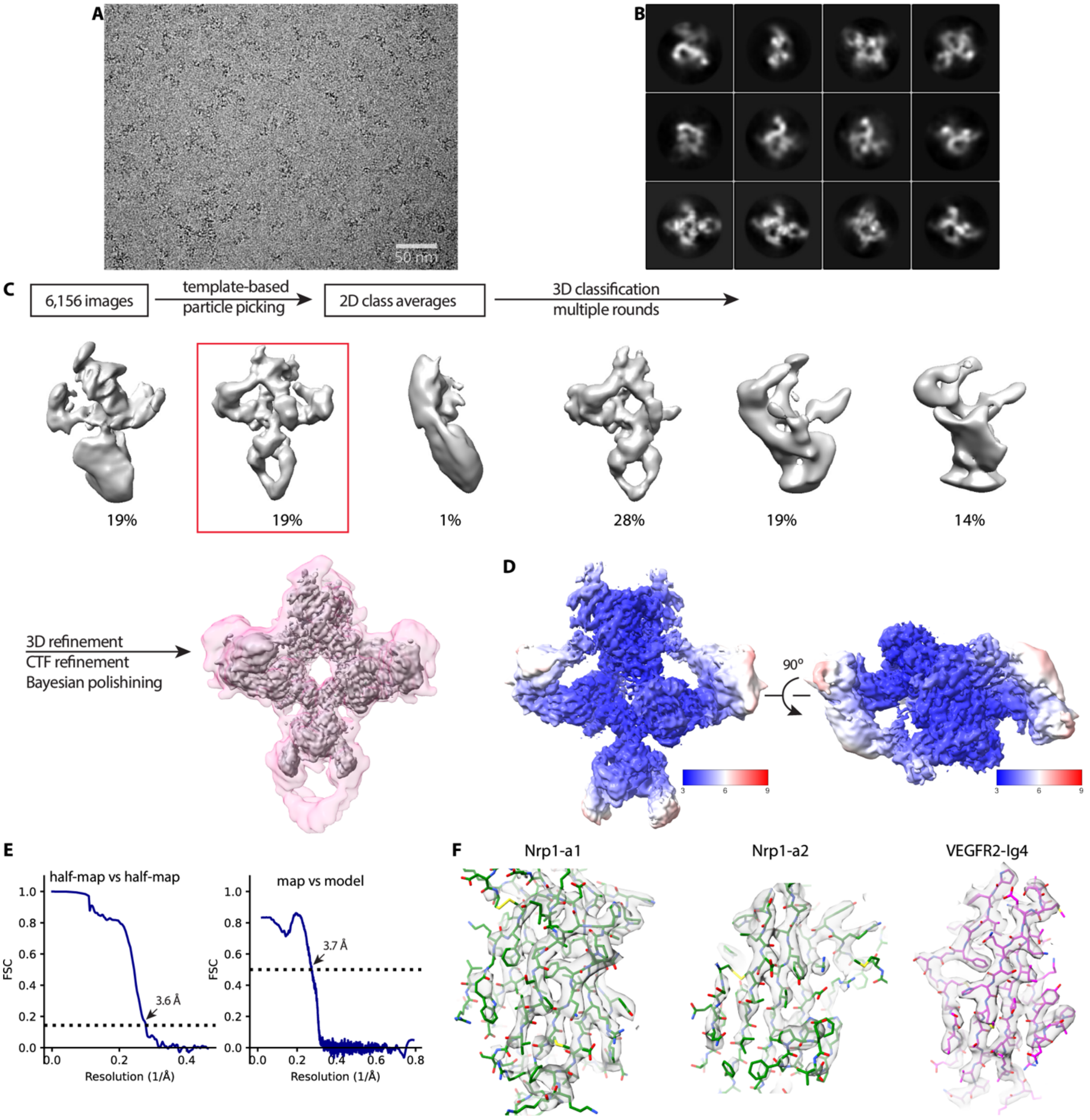

Fig. S1. Cryo-EM image-processing workflow for the $VEGF_{164}$-VEGFR2-Nrp1 dimeric complex.

(**A**) Representative micrograph. (**B**) Representative 2D class averages. (**C**) Image-processing procedure. (**D**) Final reconstruction colored according to local resolution. (**E**) FSC curves calculated between the two half-maps and between the map and the atomic model, respectively. (**F**) Sample densities of Nrp1-a1, Nrp1-a2 and VEGFR2-Ig4.

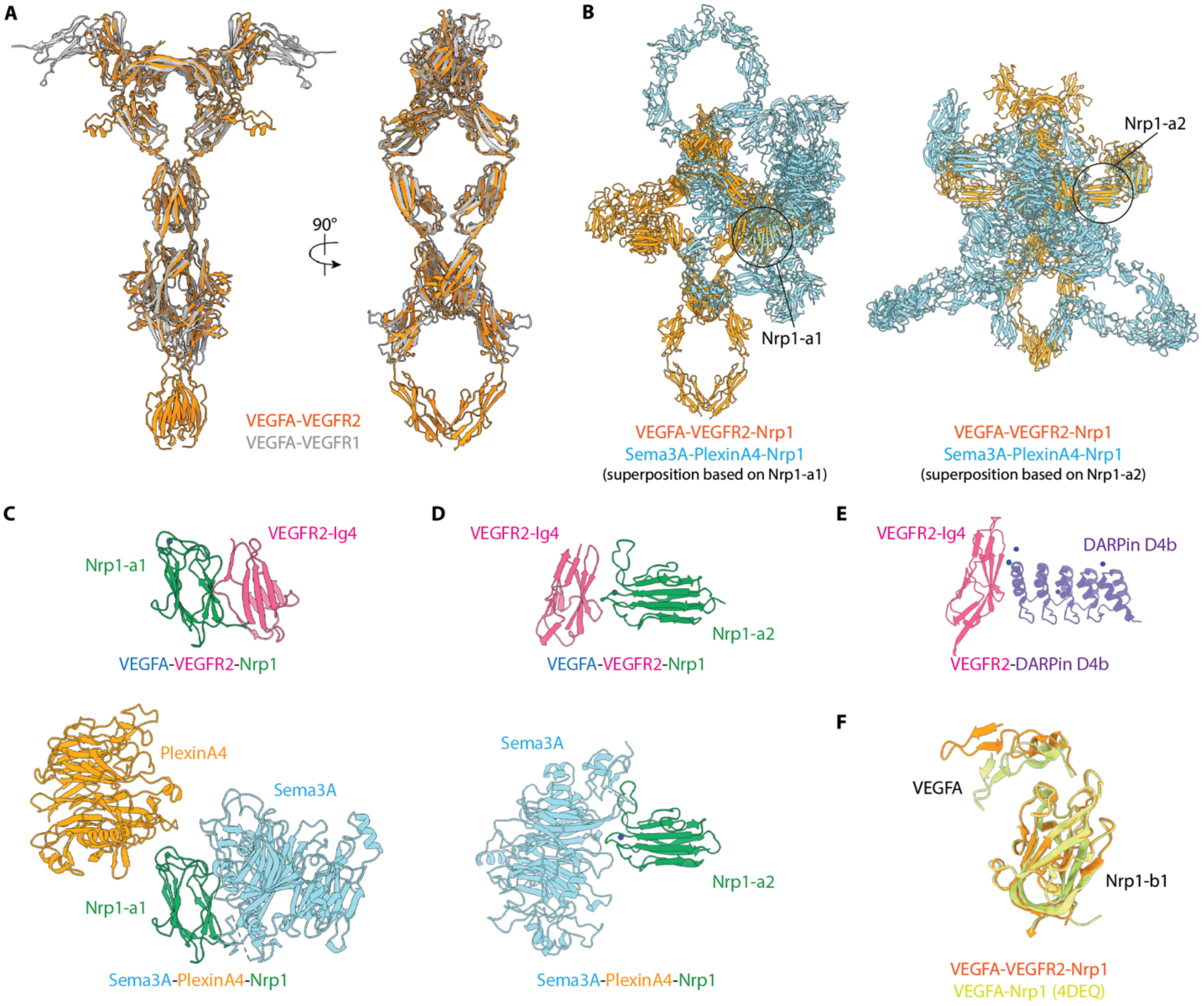

Fig. S2. Comparison of the $VEGF_{164}$-VEGFR2-Nrp1 complex structure with previously published structures of Nrp1 and VEGFR2.

(**A**) Comparison of the $VEGF_{164}$-VEGFR2 complex in our dimeric complex structure and the VEGFA-VEGFR1 complex (PDB ID: 5T89). (**B**) Superposition of the $VEGF_{164}$-VEGFR2-Nrp1 and Sema3A-PlexinA4-Nrp1 (PDB ID: 7M0R) complexes based on Nrp1-a1 (left) and Nrp1-a2 (right). (**C**) Comparison of the binding of Nrp1-a1 with VEGFR2-Ig4, PlexinA4 and Sema3A (PDB ID: 7M0R). (**D**) Comparison of the binding of Nrp1-a2 with VEGFR2-Ig4 and Sema3A (PDB ID: 7M0R). (**E**) Binding mode between VEGFR2-Ig4 and DARPin D4b (PDB ID: 5OYJ). (**F**) Comparison of the VEGF HBD and Nrp1-b1 in the $VEGF_{164}$-VEGFR2-Nrp1 structure and the VEGFA-Nrp1 complex (PDB ID: 4DEQ).

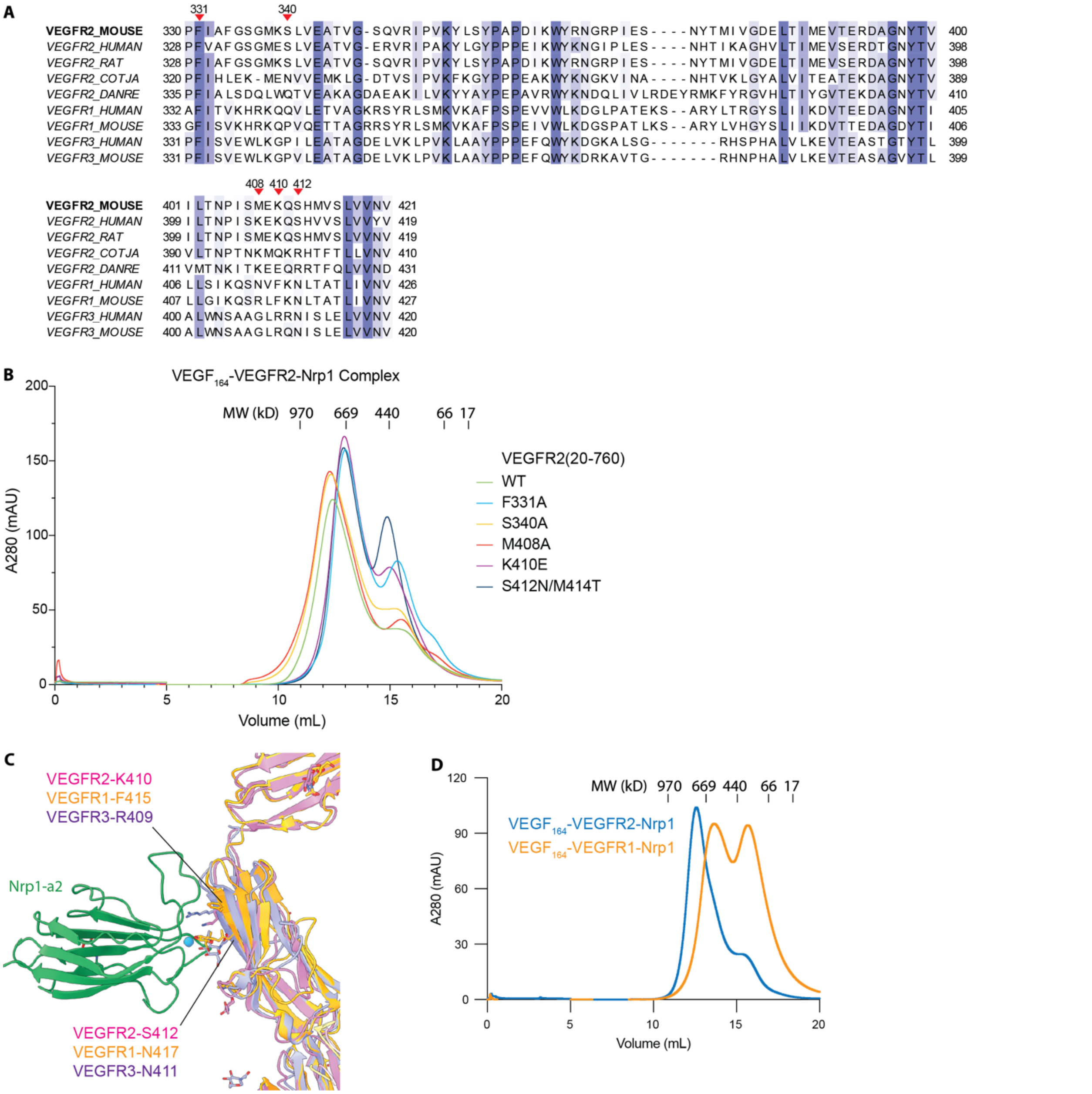

Fig. S3. Analyses of the surface of VEGFR2-Ig4 involved in the interaction with Nrp1.

(**A**) Sequence alignment of Ig4 domains in VEGFR2, VEGFR1 and VEGFR3. Residues in VEGFR2 involved in Nrp1 binding are marked with red triangles. (**B**) Effects of mutations in VEGFR2-Ig4 domain on the formation of the dimeric $VEGF_{164}$-VEGFR2-Nrp1 complex, assessed by SEC on a Superose 6 column. F331A, K410E and S412N/M414T caused a right shift of the complex peak, indicating reduced complex formation. (**C**) Structural comparison of the Ig4 domain of VEGFR1-3. The Nrp1-interacting residue K410 in VEGFR2 is not conserved in VEGFR1 or VEGFR3. Both VEGFR1 (N417) and VEGFR2 (N411) contain N-glycosylation sites at positions that would clash with Nrp1-a2. (**D**) Comparison of the SEC profiles of $VEGF_{164}$-VEGFR2-Nrp1 and $VEGF_{164}$-VEGFR1-Nrp1. The complex peak of $VEGF_{164}$-VEGFR1-Nrp1 eluted later than that of $VEGF_{164}$-VEGFR2-Nrp1, indicating that VEGFR1 does not form a stable 2:2:2 dimeric complex with $VEGF_{164}$ and Nrp1.

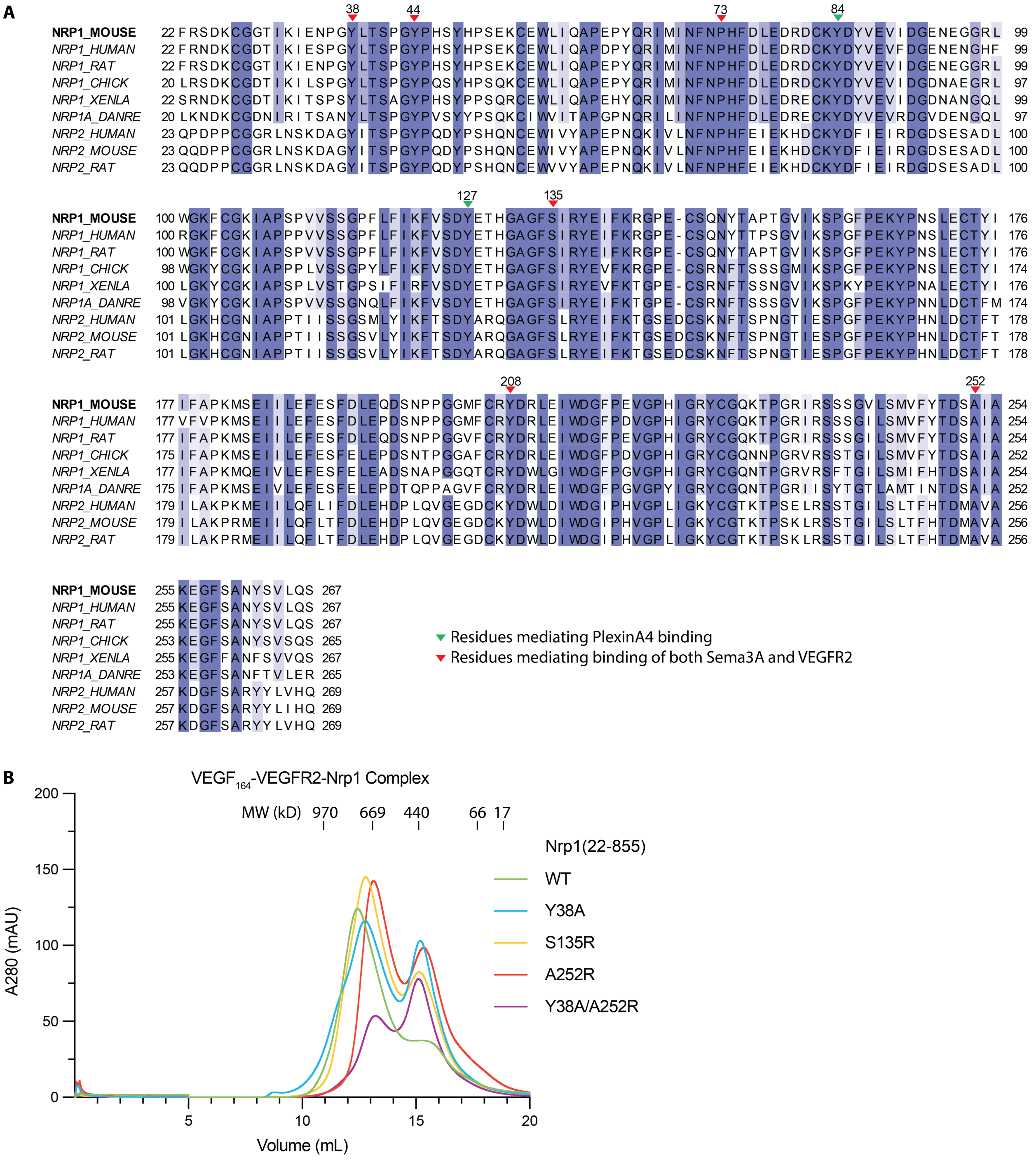

Fig. S4. Analyses of residues in Nrp1 involved in the binding interface with VEGFR2.

(**A**) Sequence alignment of the a1 and a2 domains in Nrp1 and Nrp2 from different species. (**B**) Analyses of the effects of mutations in the Nrp1 a1 and a2 domains on the formation of the $VEGF_{164}$-VEGFR2-Nrp1 complex by SEC on a Superose 6 column. These mutations caused right shifts of the peak to varying extents, indicating destabilization of the complex.

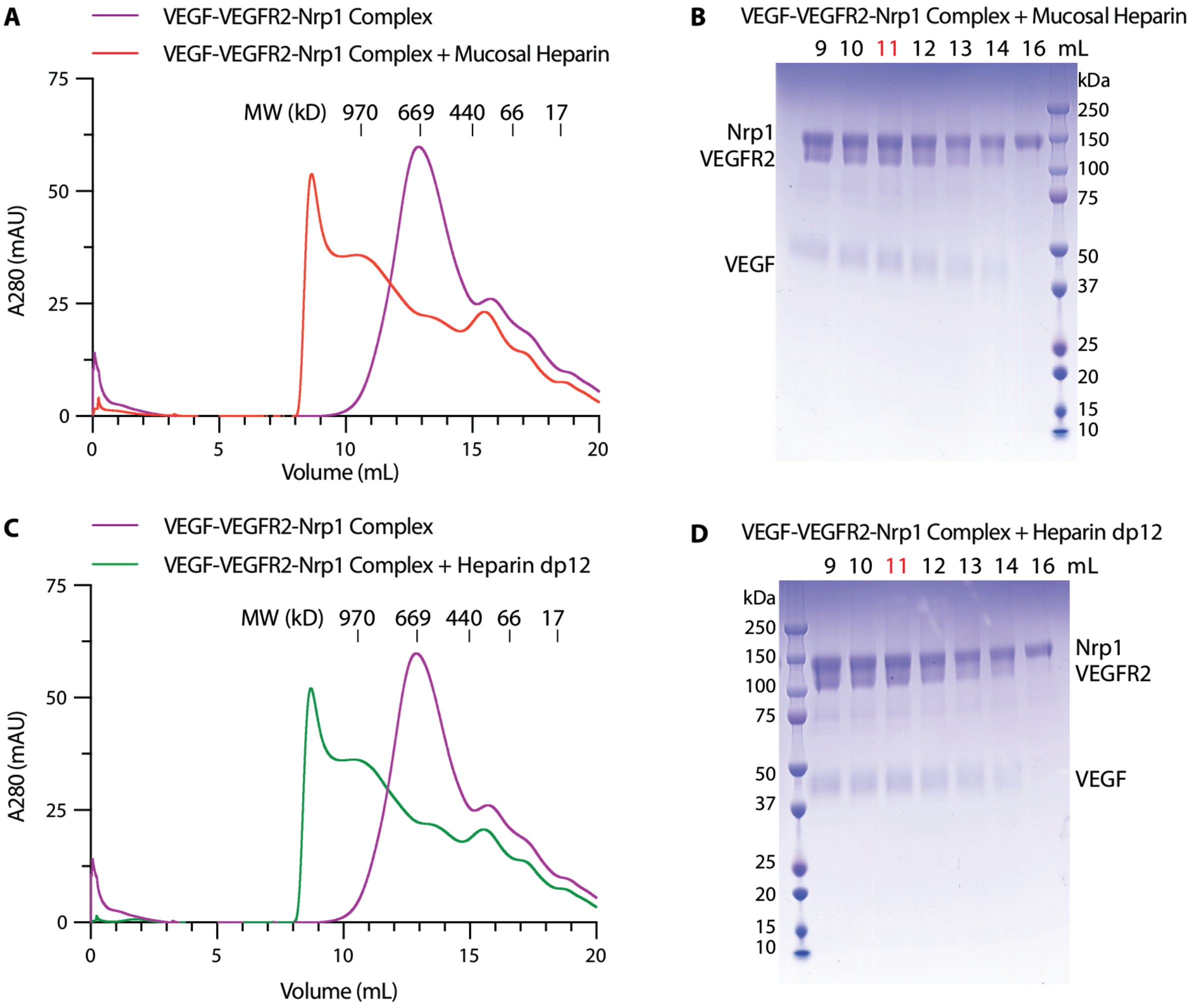

Fig. S5. High-order complex of $VEGF_{164}$, VEGFR2 and Nrp1 induced by heparin.

(**A**) Mucosal heparin induced a large left shift of the $VEGF_{164}$-VEGFR2-Nrp1 complex on Superose 6, indicating formation of a larger protein-heparin complex. (**B**) SDS-PAGE analyses of fractions of the heparin-containing complex from panel **A**. The fraction at 11 mL was used as the cryo-EM sample. (**C**) Heparin dp12 induced a large left shift of the $VEGF_{164}$-VEGFR2-Nrp1 complex on Superose 6, indicating formation of a large protein-heparin complex. (**D**) SDS-PAGE analyses of fractions the heparin-containing complex from panel **C**. The fraction at 11 mL was used as the cryo-EM sample.

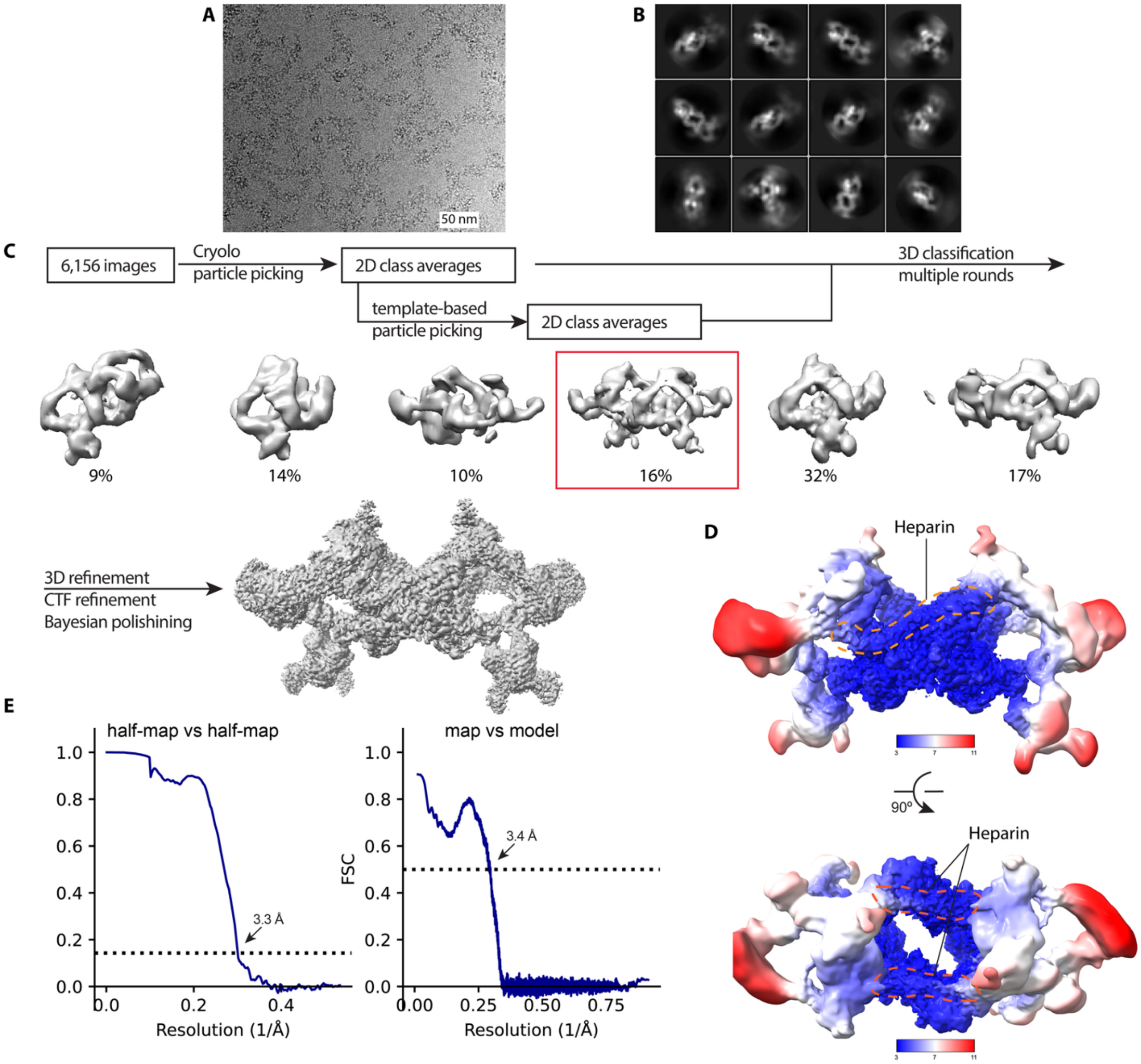

Fig. S6. Cryo-EM image-processing workflow for the cis tetrameric complex of $VEGF_{164}$, VEGFR2 and Nrp1 induced by mucosal heparin.

(**A**) Representative micrograph. (**B**) Representative 2D class averages. (**C**) Image-processing procedure. (**D**) Final reconstruction colored according to local resolution. (**E**) FSC curves calculated between the two half-maps and between the map and the atomic model, respectively.

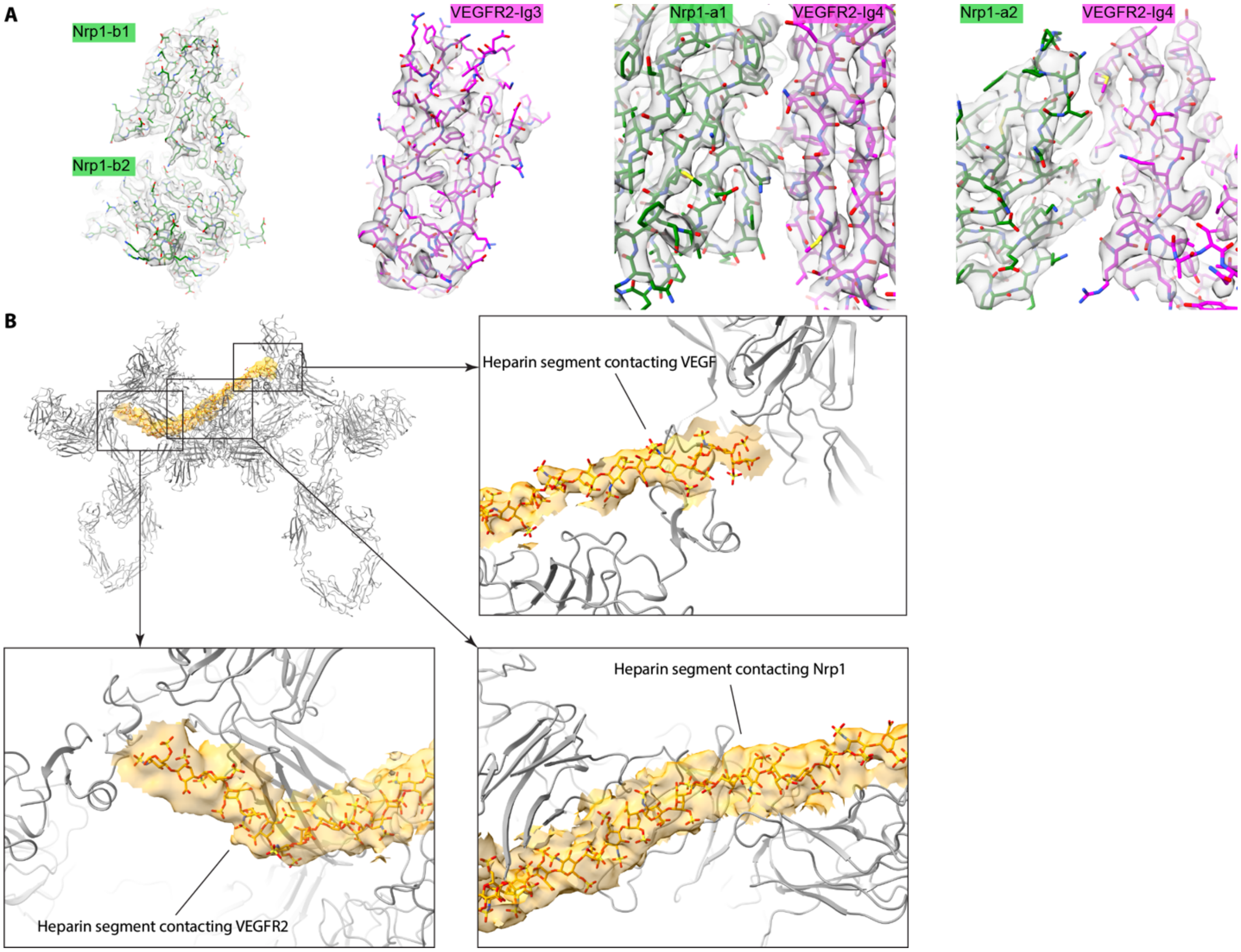

Fig. S7. Sample densities of the cis tetrameric complex of VEGF$_{164}$-VEGFR2-Nrp1 induced by mucosal heparin.

(**A**) Sample densities of Nrp1-b1, Nrp1-b2, VEGFR2-Ig3, and the interfaces between Nrp1 and VEGFR2. (**B**) Densities of different parts of the mucosal heparin molecule.

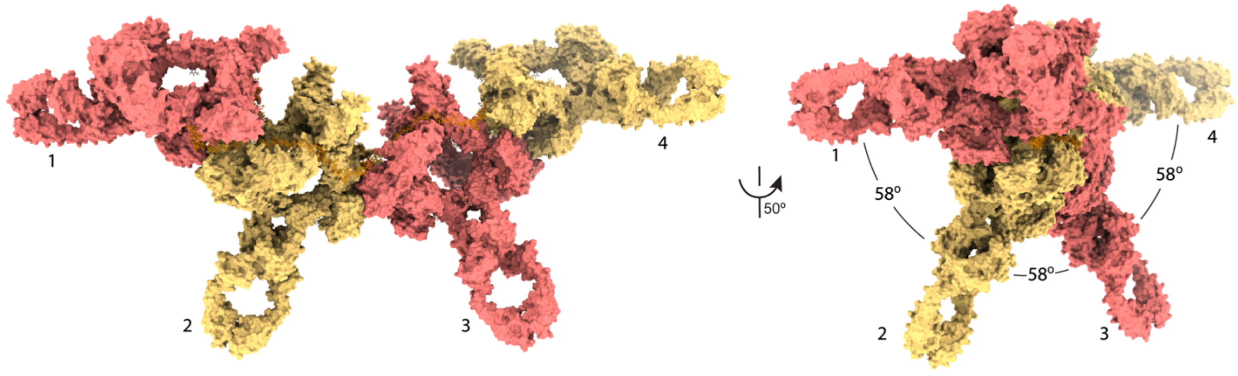

Fig. S8. Larger assembly of the VEGF$_{164}$-VEGFR2-Nrp1 complex induced by mucosal heparin.

Four dimeric complexes (labelled as 1, 2, 3 and 4) are modeled based on the cis tetrameric complex. Due to ~58°-rotation between consecutive dimeric complexes, the orientation of the fourth complex is opposite to that of the first one, suggesting that such daisy chain-like assembly is unlikely to form on the same cell surface.

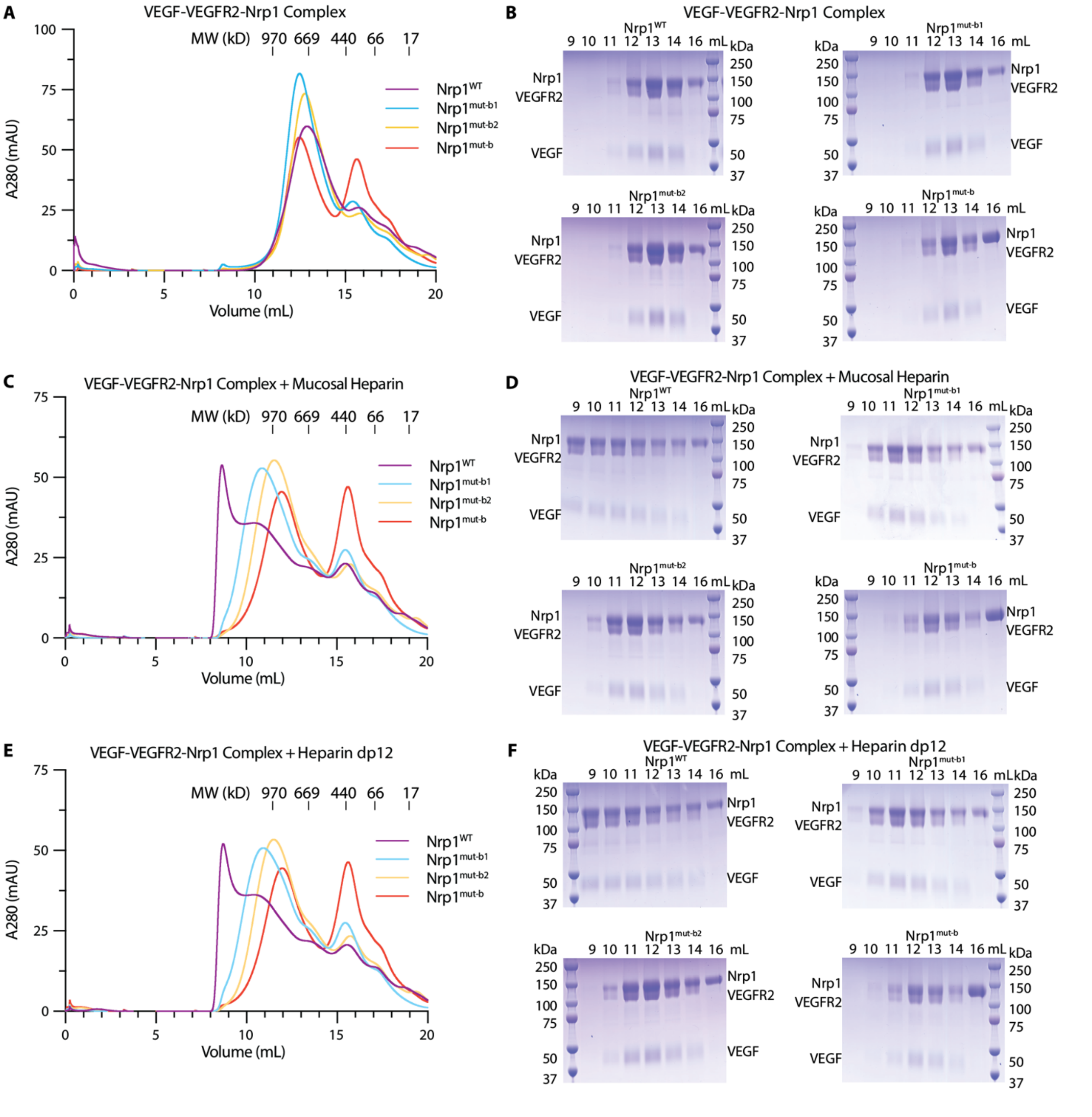

Fig. S9. Analyses of the effects of mutations in the heparin-binding sites in Nrp1 on the complex formation and high-order oligomerization of $VEGF_{164}$, VEGFR2 and Nrp1 with SEC on a Superose 6 column.

(**A** and **B**) Mutations in the heparin-binding sites in Nrp1 do not affect the dimeric complex formation in the absence of heparin. (**C** and **D**) Mutations in the heparin-binding sites in Nrp1 reduced the formation of the high-order complexes induced by mucosal heparin, as shown by later elution on the column. (**E** and **F**) Mutations in the heparin-binding sites in Nrp1 reduced the formation of the high-order complexes induced by heparin dp12, as shown by the later elution of the column.

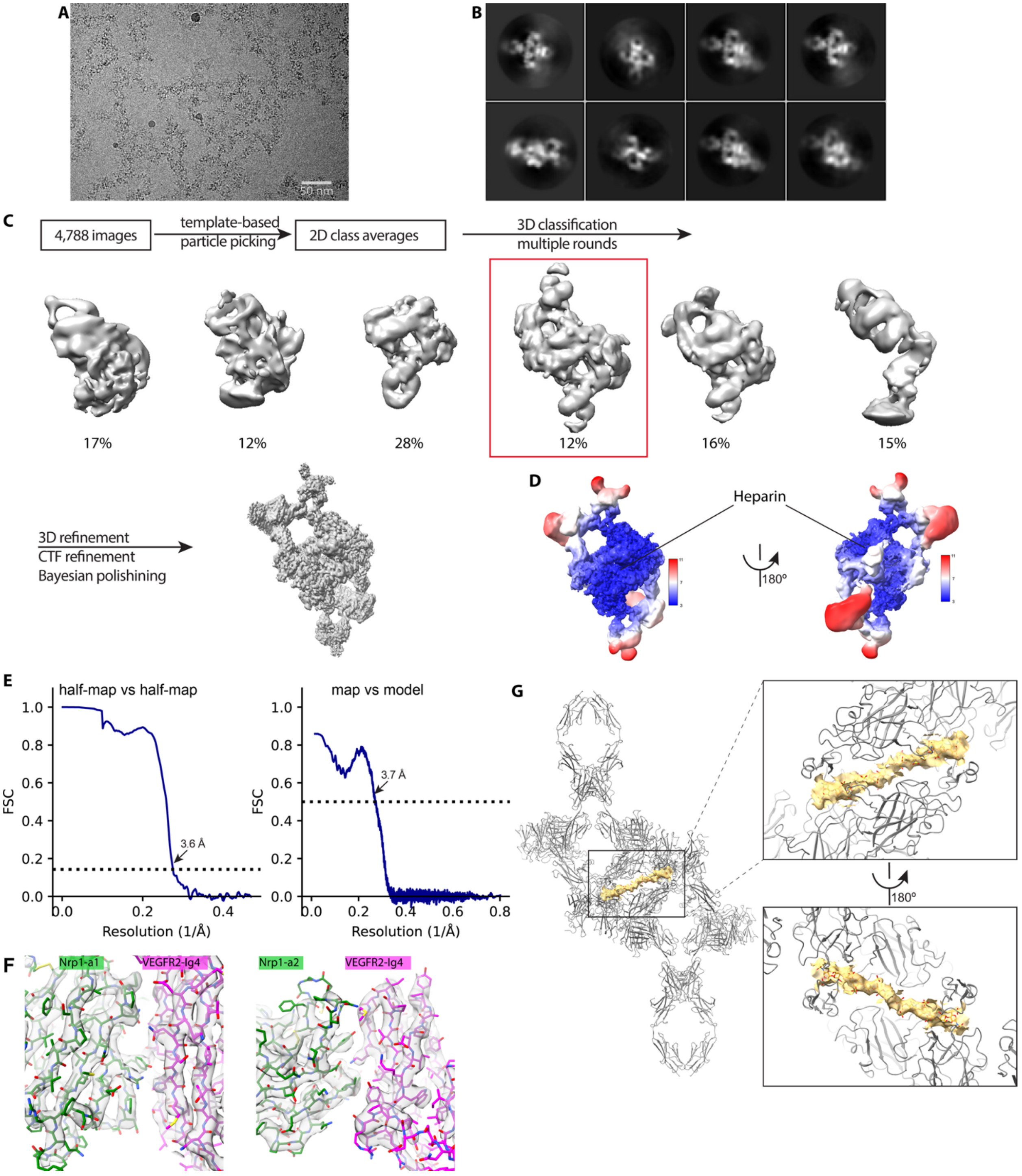

Fig. S10. Cryo-EM image-processing workflow for the trans tetrameric complex of $VEGF_{164}$, VEGFR2 and Nrp1 induced by heparin dp12.

(**A)** Representative micrograph. (**B)** Representative 2D class averages. (**C)** Image-processing procedure. (**D)** Final reconstruction colored according to local resolution. (**E)** FSC curves calculated between the two half-maps and between the map and the atomic model, respectively. (**F)** Sample densities of the binding interfaces between Nrp1 and VEGFR2. (**G)** Density of heparin dp12.

Table S1. Cryo-EM data collection and structure refinement statistics.

Structure	VEGF/VEGFR2/Nrp1<br>no heparin<br>dimeric complex	VEGF/VEGFR2/Nrp1<br>with mucosal heparin<br>cis tetrameric complex	VEGF/VEGFR2/Nrp1<br>with dp12 heparin<br>trans tetrameric complex
Magnification	81,000	130,000	81,000
Voltage (kV)	300	300	300
Electron exposure ($e^-/Å^2$)	50	50	50
Defocus range (μm)	1.0-2.5	1.5-2.5	1.0-2.5
Pixel size (Å)	1.08	0.936	1.066
Symmetry imposed	C2	C2	C2
Initial particle images (no.)	1,109,846	2,823,329	1,582,828
Final particle images (no.)	64,468	105,039	75,176
Map resolution (Å)	3.6	3.3	3.6
FSC threshold	0.143	0.143	0.143
Initial model used<br>(AlphaFold model #)	VEGF: AF-Q00731-F1<br>VEGFR: AF-P35918-F1<br>Nrp1: AF-P97333-F1	VEGF: AF-Q00731-F1<br>VEGFR: AF-P35918-F1<br>Nrp1: AF-P97333-F1	VEGF: AF-Q00731-F1<br>VEGFR: AF-P35918-F1<br>Nrp1: AF-P97333-F1
Model resolution (Å)	3.7	3.5	3.7
FSC threshold	0.5	0.5	0.5
Map sharpening B factor ($Å^2$)	-50	0	0
Model Composition			
Non-hydrogen atoms	20,326	42,270	41,492
Protein residues	2574	5,198	5196
Ligands	4 Ca^{2+}	8 Ca^{2+}<br>2 heparin chains	8 Ca^{2+}<br>2 tinzaparin chains
Protein B factors ($Å^2$)	200.0	322.0	179.4
Ligand B factors ($Å^2$)	114.3	287.9	144.1
R.m.s. deviations			
Bond length (Å)	0.004	0.002	0.004
Bond angle (°)	0.72	0.667	0.75
Validation			
Molprobity score	1.6	1.1	1.6
Clashscore	5.1	1.83	4.4
Poor rotamers (%)	0.2	0.6	0.2
Ramachandran plot			
Favored (%)	95.3	97.1	94.3
Allowed (%)	4.7	2.9	5.4
Outliers (%)	0	0	0.3
